# mHealth-Based Gamification Interventions Among Men Who Have Sex With Men in the HIV Prevention and Care Continuum: Systematic Review and Meta-Analysis

**DOI:** 10.2196/49509

**Published:** 2024-04-15

**Authors:** Qianqian Luo, Yue Zhang, Wei Wang, Tianyu Cui, Tianying Li

**Affiliations:** 1 School of Nursing, Binzhou Medical University, Yantai, China; 2 Department of Nursing, The People's Hopstial of Laoling City, Dezhou, China

**Keywords:** mHealth, gamification, HIV, men who have sex with men, meta-analysis, PRISMA, mobile health, Preferred Reporting Items for Systematic Reviews and Meta-Analyses

## Abstract

**Background:**

In the past few years, a burgeoning interest has emerged in applying gamification to promote desired health behaviors. However, little is known about the effectiveness of such applications in the HIV prevention and care continuum among men who have sex with men (MSM).

**Objective:**

This study aims to summarize and evaluate research on the effectiveness of gamification on the HIV prevention and care continuum, including HIV-testing promotion; condomless anal sex (CAS) reduction; and uptake of and adherence to pre-exposure prophylaxis (PrEP), postexposure prophylaxis (PEP), and antiretroviral therapy (ART).

**Methods:**

We comprehensively searched PubMed, Embase, the Cochrane Library, Web of Science, Scopus, and the *Journal of Medical Internet Research* and its sister journals for studies published in English and Chinese from inception to January 2024. Eligible studies were included when they used gamified interventions with an active or inactive control group and assessed at least one of the following outcomes: HIV testing; CAS; and uptake of and adherence to PrEP, PEP, and ART. During the meta-analysis, a random-effects model was applied. Two reviewers independently assessed the quality and risk of bias of each included study.

**Results:**

The systematic review identified 26 studies, including 10 randomized controlled trials (RCTs). The results indicated that gamified digital interventions had been applied to various HIV outcomes, such as HIV testing, CAS, PrEP uptake and adherence, PEP uptake, and ART adherence. Most of the studies were conducted in the United States (n=19, 73%). The most frequently used game component was gaining points, followed by challenges. The meta-analysis showed gamification interventions could reduce the number of CAS acts at the 3-month follow-up (n=2 RCTs; incidence rate ratio 0.62, 95% CI 0.44-0.88). The meta-analysis also suggested an effective but nonstatistically significant effect of PrEP adherence at the 3-month follow-up (n=3 RCTs; risk ratio 1.16, 95% CI 0.96-1.38) and 6-month follow-up (n=4 RCTs; risk ratio 1.28, 95% CI 0.89-1.84). Only 1 pilot RCT was designed to evaluate the effectiveness of a gamified app in promoting HIV testing and PrEP uptake. No RCT was conducted to evaluate the effect of the gamified digital intervention on PEP uptake and adherence, and ART initiation among MSM.

**Conclusions:**

Our findings suggest the short-term effect of gamified digital interventions on lowering the number of CAS acts in MSM. Further well-powered studies are still needed to evaluate the effect of the gamified digital intervention on HIV testing, PrEP uptake, PEP initiation and adherence, and ART initiation in MSM.

## Introduction

### Background

The HIV epidemic among men who have sex with men (MSM) has become a global concern [1]. Current evidence suggests that MSM accounted for two-thirds of all new HIV infections in the United States in 2019 [2], and systematic reviews showed that the HIV prevalence in MSM in China increased from 1.4% in 2001 to 8% in 2015 [3,4]. Given the burden of HIV, substantial efforts have been made to address the unmet needs of MSM. For instance, there is overwhelming scientific evidence of the efficacy and safety of pre-exposure prophylaxis (PrEP) and postexposure prophylaxis (PEP) to prevent HIV infection in MSM. However, studies indicate that uptake of PrEP and PEP has been suboptimal in these risk groups, especially among racial or ethnic minorities [5]. In a recent systematic review and meta-analysis, the PEP uptake was only between 4% and 6% in MSM; major obstacles included insufficient knowledge, underestimated risk of exposure to HIV, and social stigma [6]. Interventions that improve effective HIV prevention adoption among MSM are urgently needed to maximize HIV prevention benefits.

Nearly universal mobile phone ownership provides MSM an opportunity to move away from traditional ways of meeting partners to seeking sexual partners through geo–social networking applications [7]. The widespread use of smartphones also creates a unique opportunity to design innovative internet-based digital interventions for diverse MSM in a scalable manner. Internet-based digital interventions, defined as using internet-based information and communication technology (eg, mobile apps, websites, and social media) to support health [8], benefit from being implemented at a large scale with low costs and can deliver health services at the time and place chosen by users [9]. However, previous research showed that user engagement in digital interventions is suboptimal (due to, for example, high participant attrition) [10], resulting in a significant gap in intervention efficacy.

Gamification, which first emerged in 2008 and gained popularity since the 2010s, refers to applying game components such as leaderboards, points, and badges into nongame contexts [11]. Unlike serious games, which refer to full-fledged video games for health purposes, gamification is relatively open to varying situational engagement models [12]. Previous studies have been widely conducted on the use of gamification as a means to increase the initiation of desired health behaviors [13,14]. Given its increasing use in public health, gamification might also be useful for interventions to promote HIV prevention and control services. To our knowledge, two previous reviews explored how gamification was used during the HIV prevention and care continuum; the first review was conducted in 2017 and only summarized studies published between January 2016 and March 2017 [15], and the second review searched studies published in the *Journal of Medical Internet Research* and its sister journals [16], both of which would result in an incomplete study search. Moreover, to our knowledge, no meta-analysis has hitherto examined the effectiveness of gamification applied to HIV prevention and control. Therefore, it is essential to conduct an in-depth review and provide a meta-analysis combining evidence on the effectiveness of gamified HIV digital prevention interventions. Findings from this review may have important implications for HIV digital health prevention development and future research.

### Objectives

This study was divided into two parts. The first part was a systematic review of HIV prevention and control gamification with the following aims: to describe the characteristics of included studies that applied gamification, describe the various gamification elements that are commonly applied, and evaluate the methodological quality of included studies. The second part is a meta-analysis of the effectiveness of gamification applied to HIV prevention and control to assess the impact of gamification on HIV testing; condomless anal sex (CAS) reduction; and uptake of and adherence to PrEP, nonoccupational PEP (nPEP), and antiretroviral therapy (ART). It was hypothesized that MSM participating in the gamified intervention would display higher levels of HIV prevention and care than those in the control groups.

## Methods

We conducted this systematic review and meta-analysis according to the PRISMA (Preferred Reporting Items for Systematic Reviews and Meta-Analyses) 2020 guideline [17]. The PRISMA checklist is listed in Checklist 1, and the study was registered in PROSPERO on January 27, 2023 (CRD42023392193).

### Search Strategy and Identification of Studies

The electronic databases PubMed/MEDLINE, Embase, the Cochrane Library, Web of Science, Scopus, and the *Journal of Medical Internet Research* and its sister journals were searched for scientific articles published from their inception until January 15, 2024, using a combination of Medical Subject Headings (MeSH) terms and text words: *Men who have sex with men* AND *HIV/AIDS* AND *gamification/game-based learning* AND *telemedicine*. The search strategy used for each database is listed in Multimedia Appendix 1 .

### Study Selection

The full review screening was conducted by two independent reviewers who independently checked the titles and abstracts for inclusion in the review. Full-text articles were obtained for closer inspection when an article met the inclusion criteria. In addition, reference lists of retrieved articles, existing relevant systematic reviews, and all articles citing the included studies on Google Scholar were manually searched to allocate studies not identified in the database searches. Any disagreement on study selection was resolved by discussion with a third reviewer.

To be eligible for inclusion, studies were required to describe or evaluate gamified interventions for HIV prevention and control in MSM. For the systematic review, the inclusion criteria were peer-reviewed original articles that explicitly addressed the use of game elements or gamification; the intervention described involved a task specifically designed for HIV prevention and control, and at least one game element was involved in the task; the task was delivered via a digital device (eg, smartphones, tablets, or laptops); the study population should be exclusively MSM or more than 50% of the participants should be MSM; and the primary outcome should contain at least one of the following outcomes related to HIV prevention and control, namely, HIV testing, CAS, and uptake of and adherence to PrEP, nPEP, and ART for MSM living with HIV. Given the limited number of published randomized controlled trials (RCTs), we included a heterogeneous range of study designs, including controlled pre-post studies, quasi-randomized studies, cohort studies, and case-control studies. If a study included both a protocol and efficacy study, we only included the efficacy study; otherwise, we included the study protocol. The studies included in the meta-analysis represented a subset of studies in the systematic review. An additional inclusion criterion for the meta-analysis was that the study design should be an RCT. The definition of gamification by Deterding et al [18] was adopted for this review, which considers gamification as an umbrella term for using game elements (rather than full-fledged games) to improve user experience and user engagement in nongame services and applications. Studies were excluded if they were not peer-reviewed articles including letters, commentary, conference abstracts, etc; were not written in English or Chinese; were systematic reviews or meta-analyses; the full text was not available in published form; did not include a well-described intervention; exclusively used text-based technology; or involved serious or full-fledged games.

### Study Quality Assessment

Two reviewers independently assessed the quality and risk of bias of each included RCT using the revised Cochrane Risk of Bias Tool (RoB2) [19,20], which contains 5 domains, namely, bias arising from the randomization process, bias due to deviations from the intended intervention, bias due to missing outcome data, bias in outcome measurement, and bias in the selection of the reported result. The risk of bias in each domain was judged as *low risk*, *some concerns*, or *high risk*. We used the Risk of Bias in Nonrandomized Studies of Interventions (ROBINS-I) tool to assess the risk of bias in nonrandomized studies of interventions (cohort studies, case-control studies, controlled pre-post studies, and quasi-randomized studies), which contains 7 domains, and the risk of bias in each domain was judged as *low risk*, *moderate risk*, *serious risk*, *critical risk*, or *no information* [21]. Any disputes between the two reviewers were resolved through discussion with a third author. We summarized the quality evaluation results with a risk-of-bias plot by *robvis* [22]. The average score between the two authors was calculated for each study, and the weighted Cohen κ coefficient was measured to test the interrater reliability.

### Data Extraction

A structured data extraction form was developed and revised by the authors. The extracted data comprised study characteristics, participants, intervention characteristics, comparison activity, and outcomes. The study characteristics included first author, publication year, country, study design, and study setting. Participant characteristics included participant recruitment method, recruitment period, sample size, follow-up times, mean and SD of participants’ age, and HIV status. For intervention characteristics, we extracted intervention developers, intervention names if available, the number of modules in each intervention, intervention delivery modality, game components used in each intervention, and theories used to apply gamification. The primary outcomes were CAS reduction; HIV testing; and uptake of and adherence to PrEP, nPEP, and ART. Authors of the initial publications were contacted when additional information was needed. Because of multiple classifications proposed for the term gamification, the components in each digital gamified intervention were analyzed through the lens of the Octalysis gamification framework [23], which comprises 8 core drives into which various gamification elements can be placed, namely, epic meaning and calling, development and accomplishment, empowerment creativity and feedback, ownership and possession, social influence and relatedness, scarcity and impatience, unpredictability and curiosity, and loss and avoidance (Table S3-2 in Multimedia Appendix 2 [13,24-48]). Laine et al [49] mapped their interview results from schoolchildren to the Octalysis framework to better establish a digital gamification-based intervention for promoting active school transport.

### Data Synthesis and Analysis

First, data were synthesized and summarized in a narrative form assessing the characters of included studies and game components in each intervention. This qualitative review consolidated all studies that met the eligibility criteria, including those for which we could not extract data. Second, a meta-analysis was conducted to evaluate the effectiveness of gamification applied to HIV prevention and care, and variables that mediated these relationships. For each trial, we estimated the risk ratio (RR) of HIV testing; CAS; and uptake of and adherence to PrEP, PEP, and ART, comparing the intervention group and the control group together with the SE of the log RR. Random-effects summary effect estimates were obtained from DerSimonian and Laird’s [50] random-effects meta-analyses of (log) RRs and 95% CIs from each study. When studies included more than one intervention group with gamification features, they were first combined into one group following the recommendation by the Cochrane Handbook. Studies with multiple control groups were integrated into different subgroup analyses if they compared their gamified interventions to inactive and active control groups. For studies reporting outcomes at more than 1 time point, we abstracted data in each time point after randomization. Higgins *I*^2^ and 95% CI were estimated to measure between-study heterogeneity (low heterogeneity: <25%; moderate heterogeneity: 25%-75%; large heterogeneity: >75%) [51]. When heterogeneity was detected by *I*^2^ (*P*<.05), a subgroup analysis was performed to investigate the possible source of heterogeneity: study designs, type of control group, selected samples (eg, participants with different risk levels) versus unselected samples, intervention duration, and outcome diagnosis (clinical diagnosis vs self-reporting). A meta-regression analysis was conducted if the number of RCTs for each outcome of interest was >10 [52]. Potential publication bias was explored by the Egger test and contour-enhanced funnel plots. If publication bias was present, nonparametric trim-and-fill analysis was performed to explore its impact on the meta-analysis results. All *P* values were 2-sided, and *P*<.05 was considered statistically significant. All analyses were conducted using Stata 15 (Stata Corp) using “metan” commands.

### Role of Funding Source

The study funder had no role in the study design, data collection, data analysis, and result interpretation. The corresponding author had full access to all study data and held the final responsibility for the decision to submit the manuscript for publication.

### Ethics Approval

This study was approved by the medical ethics board of Binzhou Medical University (#2021-007).

## Results

### Search Results

**Figure 1.**
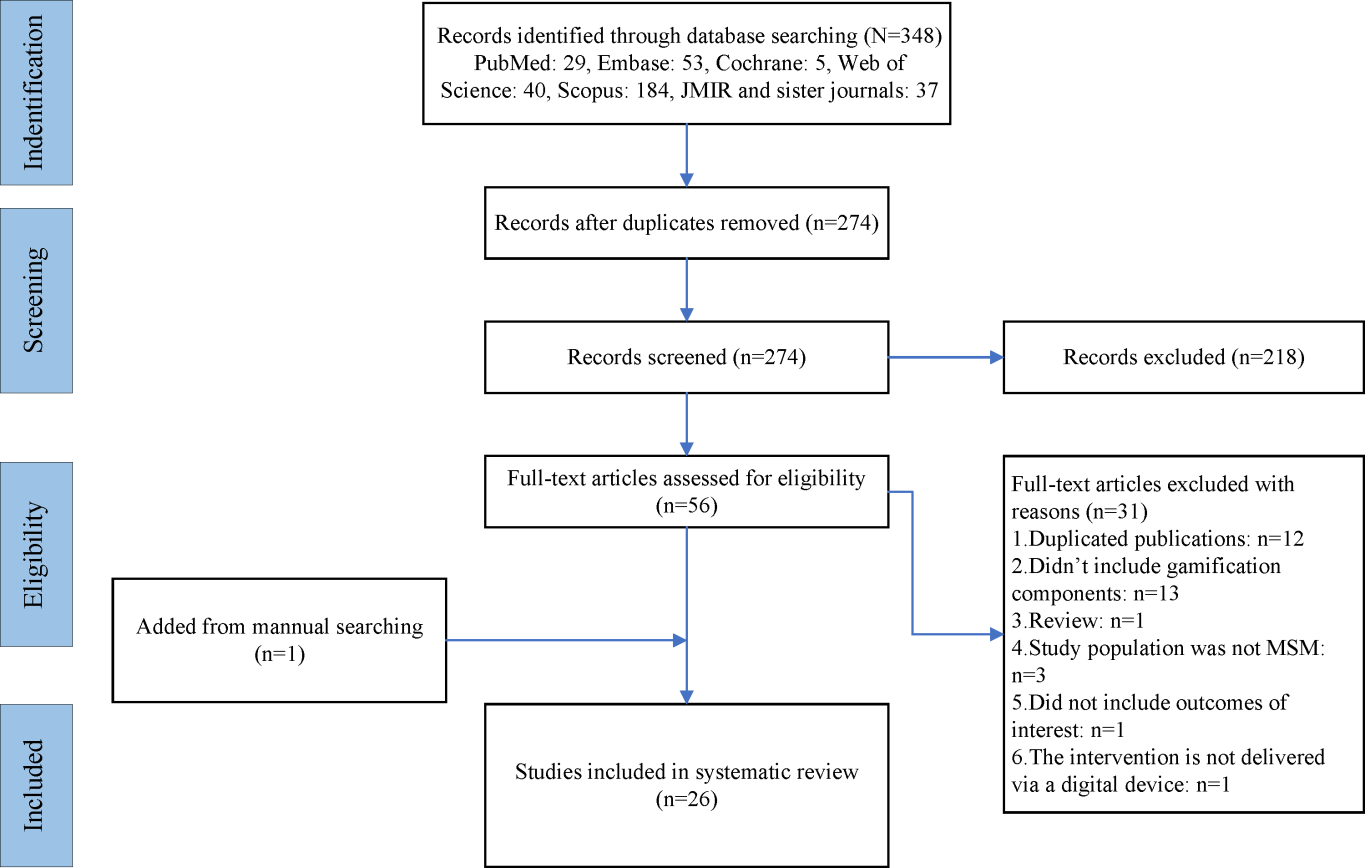
PRISMA (Preferred Reporting Items for Systematic Reviews and Meta-Analyses) flowchart of the literature-screening process. *JMIR*: *Journal of Medical Internet Research*; MSM: men who have sex with men.

Figure 1 illustrates the literature search and the screening process. A total of 348 records were initially retrieved by the database search. After removing 74 duplicates, the remaining 274 records underwent screening based on their title and abstract. Among these, 218 records were determined to be irrelevant and excluded. Next, the eligibility of 56 full-text records was assessed, yielding 25 records meeting the inclusion criteria and being included in the study. In addition, a secondary reference search was performed, yielding 1 more relevant record. Consequently, a total of 26 articles were included in the qualitative analysis [13,24-48]. Furthermore, of the 26 articles, 6 RCTs were chosen for the meta-analysis [24,29,30,32,33,39].

### Study Characteristics

The features of each included paper regarding study information, interventions, and outcomes are listed in Multimedia Appendix 2. As shown in Table 1 and Table S3-1 in Multimedia Appendix 2, the included studies were published between 2013 and 2023, with the majority published after 2018, which indicated that research on adopting gamification during the HIV prevention and care continuum among MSM is an emerging field. A total of 73% (n=19) of studies were conducted in the United States. As for study designs, most adopted an RCT design (n=10, 38%). In terms of sample sizes, the studies had a range of participants from 5 to 901. When considering all the studies included in the review, a total of 4436 participants were involved. The mean age of participants reported in the studies ranged from 16.2 to 42.7 years. Concerning primary outcomes, most studies explored the effects of gamified intervention on PrEP adherence (n=9), followed by HIV-testing promotion (n=7), changes in CAS (n=6), ART adherence (n=5), PrEP uptake (n=4), and PEP uptake (n=2).

**Table 1.** General characteristics of included studies (N=26).

Characteristics		Studies, n (%)	
**Publication year**			
	2013	1 (4)	
	2018-2019	10 (38)	
	2020-2021	9 (35)	
	2022-2023	6 (23)	
**Country**			
	United States	19 (73)	
	Malaysia	1 (4)	
	Tanzania	1 (4)	
	Indonesian	1 (4)	
	Spain	1 (4)	
	Mexico	1 (4)	
	Thailand	1 (4)	
	China	1 (4)	
**Study design**			
	Randomized controlled trial	10 (38)	
	Pre-post	5 (19)	
	Historical control design	1 (4)	
	Feasibility test	5 (19)	
	Study protocol	5 (19)	
**Main eHealth modes**			
	App	18 (69)	
	Internet	5 (19)	
	Both	2 (8)	
	Not reported	1 (4)	
**Primary outcomes**			
	Condomless anal sex reduction	6 (18)	
	HIV testing	7 (21)	
	PrEP^a^ uptake	4 (12)	
	PrEP adherence	9 (27)	
	Postexposure prophylaxis	2 (6)	
	Antiretroviral therapy adherence	5 (15)	
**Behavioral theories**			
	Motivational interviewing	1 (4)	
	Information-motivation-behavioral skills	11 (42)	
	Social cognitive theory	5 (19)	
	Integrated behavioral model	1 (4)	
	Information System Research Framework	1 (4)	
	Social learning theory	1 (4)	
	Principle of self-learning	1 (4)	
	Dyadic HIV care engagement	1 (4)	
	Levesque framework	1 (4)	
	Not reported	3 (12)	

a PrEP: pre-exposure prophylaxis.

### Gamified Digital Intervention Characteristics

As for intervention characteristics (Table S3-3 in Multimedia Appendix 2), the number of intervention modules ranged from 3 to 13. Of the 26 studies, 22 (85%) included members of the target population (MSM) during the developmental phase of the intervention. Gamification was delivered mostly by mobile apps (n=18, 69%), followed by the internet (n=5, 19%). The duration of study follow-ups ranged from 2 weeks to 15 months. Of the 26 studies, 88% (n=23) used theories for gamified intervention development. Information-motivation-behavioral skills (IMB) were used in 42% (n=11) of studies, social cognitive theory in 19% (n=5), motivational interviewing in 4% (n=1), integrated behavioral model in 4% (n=1), Information System Research Framework in 4% (n=1), social learning theory in 4% (n=1), principle of self-learning in 4% (n=1), dyadic HIV care engagement in 4% (n=1), and Levesque framework in 4% (n=1). Studies included in the systematic review used a range of game core drives and game components (Table 2 and Table S3-3 in Multimedia Appendix 2). The most frequently used game core drives were ownership and possession, followed by social influence and relatedness, development and accomplishment, empowerment creativity and feedback, and unpredictability and curiosity. The most frequently used game components were points, followed by challenge, discussion forums, and mentorship or character narrative.

**Table 2.** Type of game core drives and game components used in the included studies (N=26).

Game core drives		Studies, n (%)
**Development and accomplishment**		11 (42)
	Challenge	9 (35)
	Bottom line	1 (4)
	Leaderboard	1 (4)
**Empowerment creativity and feedback**		6 (23)
	Tailored message	2 (8)
	Online timely feedback	2 (8)
	Progress bar	2 (8)
**Ownership and possession**		17 (65)
	Points	10 (38)
	Money	1 (4)
	In-game currency	2 (8)
	Badges	5 (19)
**Social influence and relatedness**		13 (50)
	Mentorship/character narrative	6 (23)
	Discussion forum	6 (23)
	Allegiance	1 (4)
**Unpredictability and curiosity**		3 (12)
	Unpredictable incentives	2 (8)
	Gumball/bonus draws	2 (8)

### Quality Assessments of Included Studies

An overview of the different risks of bias in each study is presented in Tables S4-1 and S4-2 in Multimedia Appendix 3 [13,24,25,28-30,32-36,39,42,43,45,48]. The weighted Cohen κ coefficient was 0.88, suggesting good agreement between raters [53]. Overall, 10 RCTs were assessed using the RoB2, with 6 rated as having a high risk of bias. A high risk of bias occurred in the domains outcome measurement [24,28,30,42] and missing outcome data [29,43] (Figure S4-1 in Multimedia Appendix 3). Of the 6 nonrandomized studies assessed using the ROBINS-I tool, 2 presented a serious risk of bias [34,35] and 4 a critical risk of bias [25,36,45,48] (Figure S4-2 in Multimedia Appendix 3). These nonrandomized studies were not further included in the meta-analysis. We did not evaluate the study quality of the other 10 studies [26,27,31,37,38,40,41,44,46,47], as they were designed as study protocols or feasibility studies that did not report results for the outcomes of interest in our review.

### Effects of Gamification on CAS

**Figure 2.**
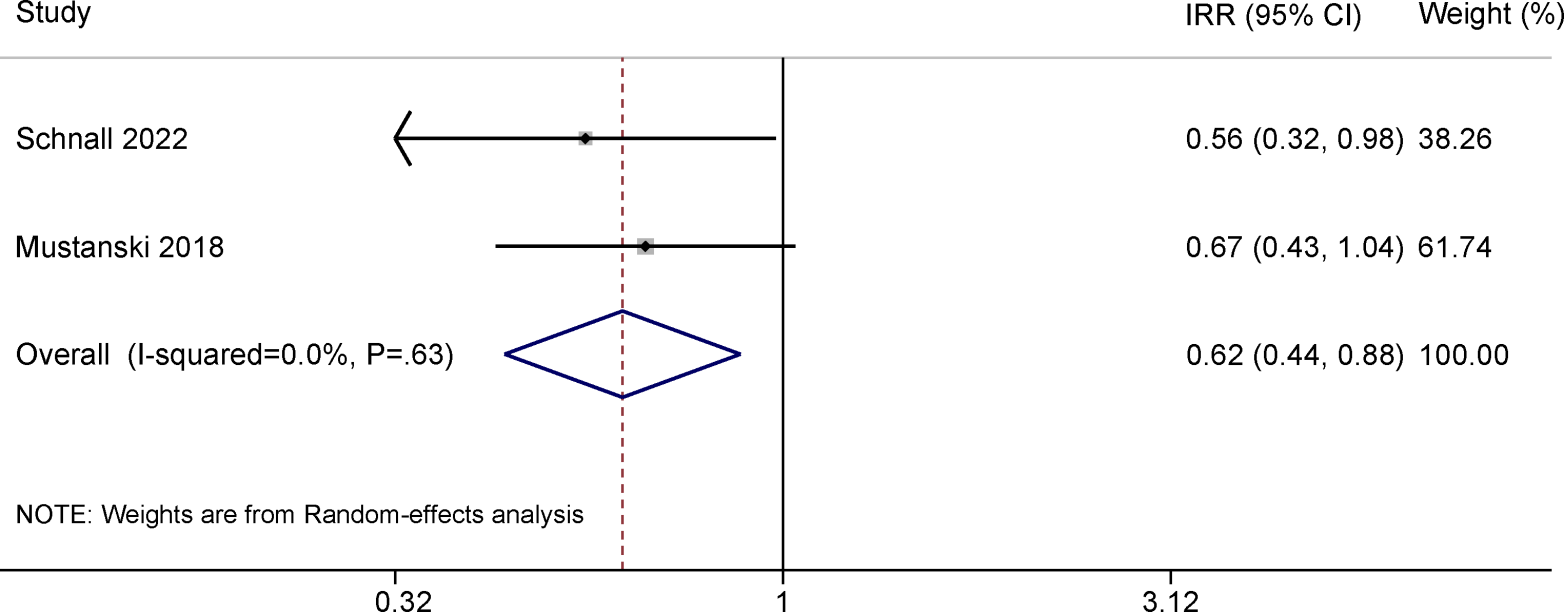
Meta-analysis of randomized controlled trials assessing the effect of gamified digital interventions on the number of condomless anal sex acts among men who have sex with men 3 months post intervention [32,33]. IRR: incidence rate ratio.

**Figure 3.**
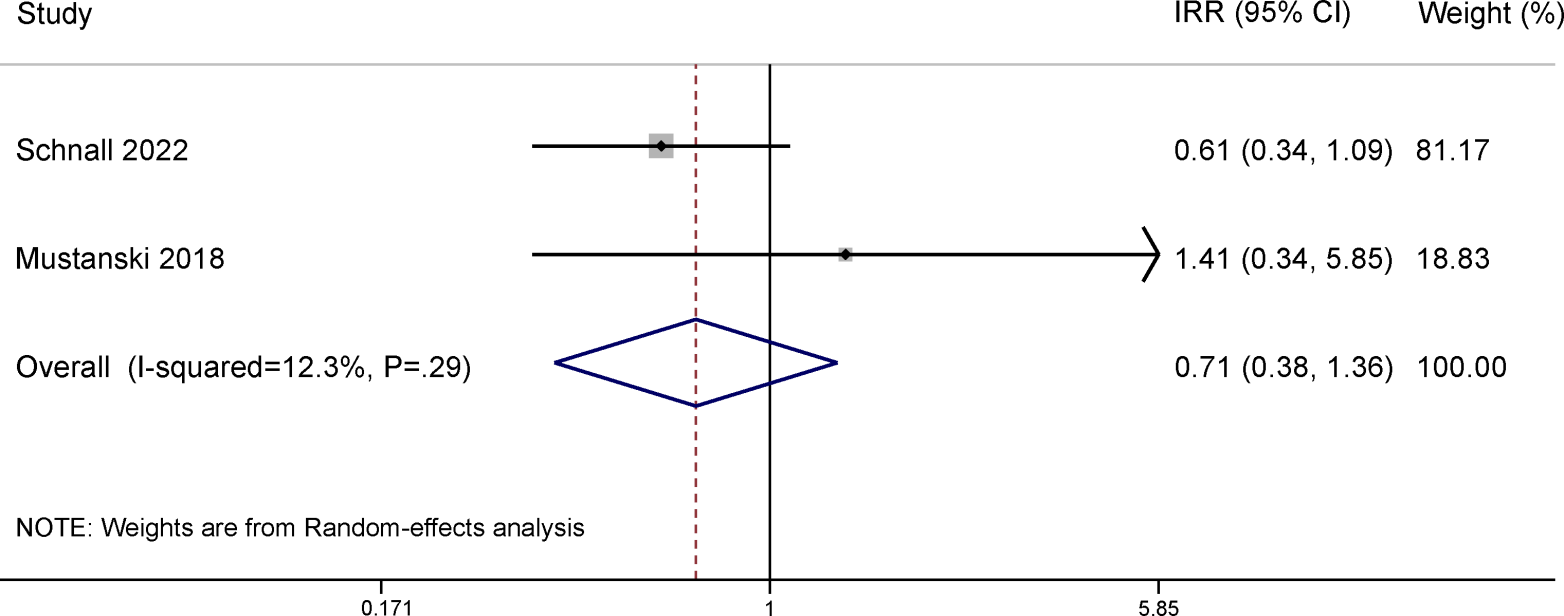
Meta-analysis of randomized controlled trials assessing the effect of gamified digital interventions on the number of condomless anal sex acts among men who have sex with men 6 months post intervention [32,33]. IRR: incidence rate ratio.

The 10 RCTs under each outcome of interest in this review are listed in Table S5-1 in Multimedia Appendix 4 [13,24-39,41-48]. The meta-analysis on the effect of gamification on CAS reduction contained 3 RCTs (total N=2138). One RCT reported the effect of gamification on engaging in CAS [43], while the other 2 evaluated the effect of gamification on the number of CAS acts and were included in the meta-analysis [32,33]. The gamified digital intervention conferred significant protection against self-reported numbers of CAS acts at the 3-month follow-up (Figure 2; incidence rate ratio [IRR] 0.62, 95% CI 0.44-0.88). However, this effect was not statistically significant 6 months post intervention (Figure 3; IRR 0.71, 95% CI 0.38-1.36). We did not find evidence of publication bias (Figure S6-1 in Multimedia Appendix 5).

### Effects of Gamification on PrEP Adherence

**Figure 4.**
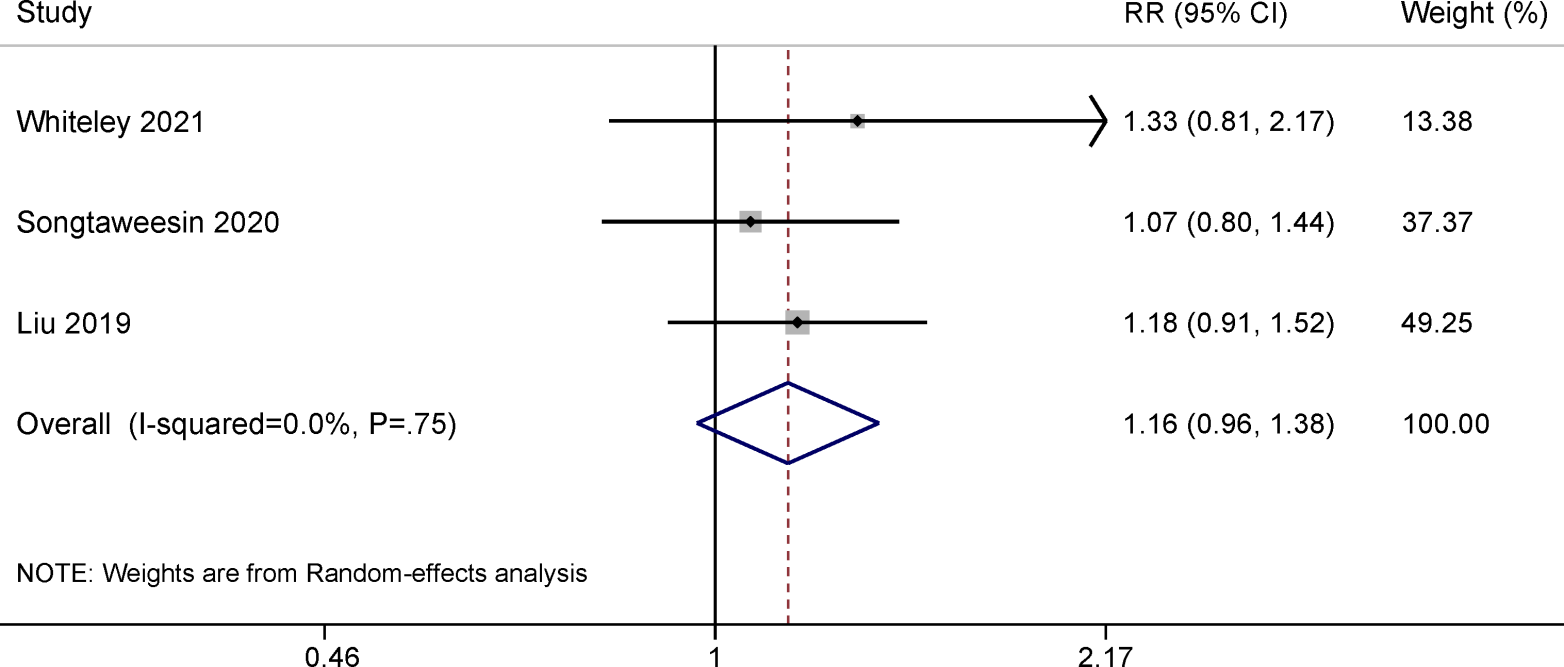
Meta-analysis of randomized controlled trials assessing the effect of gamified digital interventions on pre-exposure prophylaxis adherence among men who have sex with men at the 3-month follow-up [29,30,39]. RR: risk ratio.

**Figure 5.**
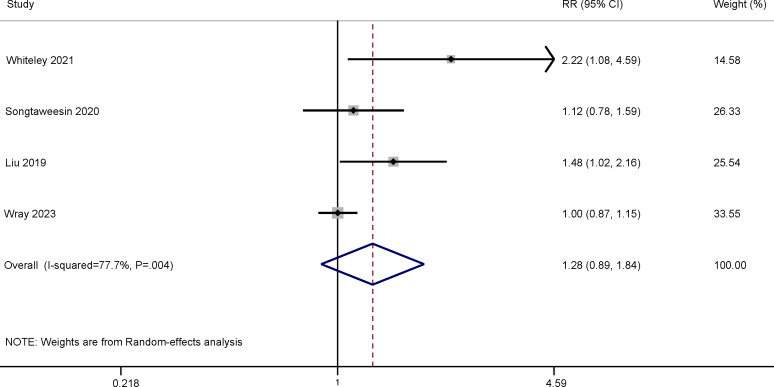
Meta-analysis of randomized controlled trials assessing the effect of gamified digital interventions on pre-exposure prophylaxis adherence among men who have sex with men at the 6-month follow-up [24,29,30,39]. RR: risk ratio.

A total of 4 RCTs compared PrEP adherence in MSM receiving a gamified digital intervention to non–PrEP-related mobile games [29], youth-friendly services [30], the standard of care from a health educator and a study clinician [39], or educational videos on sleep hygiene and diet [24]. The meta-analysis suggested an effective but nonstatistically significant effect of PrEP adherence at the 3-month follow-up (Figure 4; RR 1.16, 95% CI 0.96-1.38) and 6-month follow-up (Figure 5; RR 1.28, 95% CI 0.89-1.84). We did not find evidence of publication bias (Figure S6-1 in Multimedia Appendix 5).

Only 1 RCT was designed to evaluate a gamified mobile app (MyChoices) to increase HIV testing and PrEP uptake, and the results showed that the MyChoices arm had a 22% higher prevalence of HIV testing over the 6-month follow-up compared to those in the standard of care arm, while there was no difference in PrEP uptake between the different intervention groups [28]. Two RCTs were implemented to evaluate the effects on ART adherence among MSM living with HIV [13,42]. They used different questions to measure ART adherence, and we did not conduct a further meta-analysis for this outcome. For example, the study conducted by Hightow-Weidman et al [13] showed that the proportion of individuals self-reporting ≥90% adherence in the past 7 days rose markedly at 13 weeks post intervention with no significant difference between study arms. Horvath et al [42] evaluated an online social support intervention (Thrive With Me [TWM]) with an RCT design, and the result did not show a significant group difference for the overall ART adherence (≥90% ART adherence in the past 30 days 1 month post intervention). We found that no RCT was conducted to evaluate the effect of the gamified digital intervention on PEP uptake, PEP adherence, and ART initiation. Further studies are still needed to evaluate the effect of the gamified digital intervention on PEP initiation, PEP adherence, and ART uptake in MSM. A meta-regression analysis was not conducted since the number of RCTs in each outcome of interest was fewer than 10.

## Discussion

### Principal Findings

This study aimed to conduct a systematic review and meta-analysis to evaluate the effectiveness of gamification on the HIV prevention and care continuum among MSM. A total of 26 studies involving 10 RCTs were included in the systematic review. Our findings revealed that gamification of HIV prevention and control addressed HIV-specific outcomes, such as CAS, HIV testing, PrEP uptake, PrEP adherence, nPEP uptake, and ART adherence in MSM living with HIV, with most RCTs focusing on PrEP adherence (4/10, 40%), followed by CAS (3/10, 30%). The majority of studies were published after 2018 (25/26, 96%) and conducted in the United States (19/26, 73%), and only 6 studies were conducted in low- and middle-income countries (Malaysia, Tanzania, Indonesia, Mexico, Thailand, and China), indicating that the gamification of the HIV prevention and care continuum is an area under development. Our review showed that the most frequently used game core drive was ownership and possession, and points emerged as a frequently used game component. Besides, the most used theory in gamified digital intervention development was IMB. Overall, results from the meta-analysis of RCTs showed significant positive effects of gamification on reducing the number of CAS acts and improving PrEP adherence in MSM. However, the robustness of these findings was limited by the small number of studies included in the meta-analysis.

### Gamification Being Applied in the HIV Prevention and Care Continuum

Gamification is a technique that uses game components to motivate participants to engage positively in healthy behaviors. Cugelman [12] identified 7 game components that are linked to proven behavior change strategies, including goal setting, offering a challenge, feedback, reinforcement, compare progress, social connectivity, and fun and playfulness. In this review, we found the most frequently used game element was reinforcement (gaining points), followed by the capacity to overcome a challenge, which is consistent with reviews in other health areas, such as physical activity [54] and cognitive training [55]. Reinforcement represents the motivation driven by feelings of owning something and the desire to get more of it. Hence, healthy behaviors are mostly reinforced by the desire for possession. Challenge is a game component that falls under the core drive development and accomplishment, which motivates individuals through personal growth and the drive to achieve specific targeted goals [23]. Although the above game elements have been widely used in HIV prevention and care interventions, other game elements are less reported in research studies, such as unpredictability and curiosity, loss and avoidance, and scarcity and impatience. Hence, researchers must collaborate with companies focusing on gamification technology to include different game components for the future development of digital interventions.

### Effects of Gamification on CAS

The meta-analysis of 2 RCTs demonstrated a 38% reduction in the number of CAS acts in the intervention group compared with the control group at the 3-month follow-up. In addition, a 29% reduction in the number of CAS acts was reported at the 6-month follow-up, with no statistical significance (IRR 0.71, 95% CI 0.38-1.36), which suggests a short-term intervention effect. These findings have important implications, as all of these RCTs enrolled young MSM (ages 13-29 years) disproportionately affected by HIV [56], and few HIV prevention interventions were targeted for young MSM [57]. Moreover, given that mobile technology provides greater access to the target population who may not be able or willing to come in person to receive HIV health service, it provides an ideal platform to reach these young gay, bisexual, and transgender individuals whose ownership of smartphones and use of mobile phone apps are increasing.

### Effects of Gamification on HIV PrEP Adherence

Our pooled results indicated that gamified digital interventions effectively improved PrEP adherence in MSM. At the 3-month follow-up, those in the intervention group were 1.16 times more likely to engage in optimal PrEP dosing (≥700 fmol/punch measured by dried blood spots) than the control. The proportion of participants who engaged in optimal PrEP dosing was 28% higher in the intervention versus control groups at the 6-month follow-up. Similar results on behavior change have also been found in asthma and vaccination uptake [58,59]. Notably, in the development stage of their interventions, 3 of the 4 RCTs adopted the IMB theory, which is a behavioral change theory that may explain the effectiveness of these interventions [29,30,39]. Researchers have also suggested that interventions guided by theories are more efficient than those not driven by theories [60]. The IMB model asserts that informed and motivated participants with adequate skills for enacting adherence-related behaviors would optimally adhere to their PrEP regimen over time [61]. Interventions that integrate gamification components can educate users to interface in a more dynamic, immersive, and engaging way to make the experience more informative and motivating for individuals to promote positive health behavior change.

Some concerns should be mentioned after this review. First, more than half of studies on gamification were conducted in the United States, limiting generalizability to other countries. Second, the intervention module and duration were different in different interventions. For example, MSM had access to TWM for 5 months [62], while participants only received access to MyPEEPS over 3 months [32]. Even in an assigned intervention duration, participants’ use patterns for different intervention components varied [39]. Reasons may include a lack of ongoing new features embedded in apps, and the effectiveness may be not sustained long-term if the gamification is not continuously improved.

### Study Limitations

There were some limitations in this review. First, the number of RCTs available for each outcome of interest in the meta-analysis was limited, suggesting the need for further validation of our findings through additional studies. Second, as previous studies established that intervention engagement metrics (eg, frequency, duration, or amount of mobile health [mHealth] use) were positively associated with interested outcomes [13,63], we were unable to conduct an in-depth analysis of participants’ electronic paradata and how these data could affect our result because of the small size of studies. Indeed, further trials are needed to better measure the effective mediators in gamification for healthy behavior. Additionally, the effects of gamification on CAS and PrEP adherence were only pooled 3 and 6 months post intervention. Accordingly, little is currently known about the longer-term impact of the gamified digital intervention.

### Conclusions

This study indicated that mHealth-based gamification interventions in the HIV prevention and care continuum among MSM are in a phase of rapid evolution and continued development. Our study substantiated the short-term effect of gamification on CAS in MSM, although the long-term impact of gamified digital interventions remains to be determined.

## Supplementary material

10.2196/49509 Multimedia Appendix 1 Search strategy.

10.2196/49509 Multimedia Appendix 2 Summary of studies included in the review.

10.2196/49509 Multimedia Appendix 3 Study quality assessment.

10.2196/49509 Multimedia Appendix 4 Study effects.

10.2196/49509 Multimedia Appendix 5 Publication bias.

10.2196/49509 Checklist 1 The PRISMA (Preferred Reporting Items for Systematic Reviews and Meta-Analyses) checklist.

## Abbreviations

ART: antiretroviral therapy
CAS: condomless anal sex
IMB: information-motivation-behavioral skills
IRR: incidence rate ratio
MeSH: Medical Subject Headings
mHealth: mobile health
MSM: men who have sex with men
nPEP: nonoccupational postexposure prophylaxis
PEP: postexposure prophylaxis
PrEP: pre-exposure prophylaxis
PRISMA: Preferred Reporting Items for Systematic Reviews and Meta-Analyses
RCT: randomized controlled trial
RoB2: Risk of Bias Tool
ROBINS-I: Risk of Bias in Nonrandomized Studies of Interventions
RR: risk ratio
TWM: Thrive With Me

## References

[1] Mayer KH, Bekker LG, Stall R, Grulich AE, Colfax G, Lama JR (2012). Comprehensive clinical care for men who have sex with men: an integrated approach. Lancet.

[2] Pitasi MA, Beer L, Cha S (2021). Vital signs: HIV infection, diagnosis, treatment, and prevention among gay, bisexual, and other men who have sex with men - United States, 2010-2019. MMWR Morb Mortal Wkly Rep.

[3] Chow EPF, Wilson DP, Zhang J, Jing J, Zhang L (2011). Human immunodeficiency virus prevalence is increasing among men who have sex with men in China: findings from a review and meta-analysis. Sex Transm Dis.

[4] Tang S, Tang W, Meyers K, Chan P, Chen Z, Tucker JD (2016). HIV epidemiology and responses among men who have sex with men and transgender individuals in China: a scoping review. BMC Infect Dis.

[5] Kanny D, Jeffries WL, Chapin-Bardales J (2019). Racial/ethnic disparities in HIV preexposure prophylaxis among men who have sex with men - 23 urban areas, 2017. MMWR Morb Mortal Wkly Rep.

[6] Wang Z, Yuan T, Fan S (2020). HIV nonoccupational postexposure prophylaxis among men who have sex with men: a systematic review and meta-analysis of global data. AIDS Patient Care STDS.

[7] Phillips G 2nd, Magnus M, Kuo I (2014). Use of geosocial networking (GSN) mobile phone applications to find men for sex by men who have sex with men (MSM) in Washington, DC. AIDS Behav.

[8] Burke LE, Ma J, Azar KMJ (2015). Current science on consumer use of mobile health for cardiovascular disease prevention: a scientific statement from the American Heart Association. Circulation.

[9] Muessig KE, Nekkanti M, Bauermeister J, Bull S, Hightow-Weidman LB (2015). A systematic review of recent smartphone, internet and web 2.0 interventions to address the HIV continuum of care. Curr HIV/AIDS Rep.

[10] McCoy SI, Packel L (2020). Lessons from early stage pilot studies to maximize the impact of digital health interventions for sexual and reproductive health. Mhealth.

[11] Zainuddin Z, Chu SKW, Shujahat M, Perera CJ (2020). The impact of gamifcation on learning and instruction: a systematic review of empirical evidence. Educa Res Rev.

[12] Cugelman B (2013). Gamification: what it is and why it matters to digital health behavior change developers. JMIR Serious Games.

[13] Hightow-Weidman L, Muessig KE, Egger JR, Vecchio A, Platt A (2021). Epic allies: a gamified mobile app to improve engagement in HIV care and antiretroviral adherence among young men who have sex with men. AIDS Behav.

[14] Lister C, West JH, Cannon B, Sax T, Brodegard D (2014). Just a fad? Gamification in health and fitness apps. JMIR Serious Games.

[15] Hightow-Weidman LB, Muessig KE, Bauermeister JA, LeGrand S, Fiellin LE (2017). The future of digital games for HIV prevention and care. Curr Opin HIV AIDS.

[16] Warsinsky S, Schmidt-Kraepelin M, Rank S, Thiebes S, Sunyaev A (2021). Conceptual ambiguity surrounding gamification and serious games in health care: literature review and development of game-based intervention reporting guidelines (GAMING). J Med Internet Res.

[17] Page MJ, McKenzie JE, Bossuyt PM (2021). The PRISMA 2020 statement: an updated guideline for reporting systematic reviews. BMJ.

[18] Deterding S, Sicart M, Nacke L, O’Hara K, Dixon D Gamification. Using game-design elements in non-gaming contexts.

[19] Sterne JAC, Savović J, Page MJ (2019). RoB 2: a revised tool for assessing risk of bias in randomised trials. BMJ.

[20] Higgins JPT, Altman DG, Gøtzsche PC (2011). The Cochrane collaboration’s tool for assessing risk of bias in randomised trials. BMJ.

[21] Sterne JA, Hernán MA, Reeves BC (2016). ROBINS-I: a tool for assessing risk of bias in non-randomised studies of interventions. BMJ.

[22] McGuinness LA, Higgins JPT (2021). Risk-of-bias visualization (Robvis): an R package and shiny web app for visualizing risk-of-bias assessments. Res Synth Methods.

[23] Chou YK (2014). Actionable Gamification: Beyond Points, Badges, and Leaderboards.

[24] Wray TB, Chan PA, Kahler CW, Ocean EMS, Nittas V (2024). Pilot randomized controlled trial of game plan for PrEP: a brief, web and text message intervention to help sexual minority men adhere to PrEP and reduce their alcohol use. AIDS Behav.

[25] Weitzman PF, Zhou Y, Kogelman L, Rodarte S, Vicente SR, Levkoff SE (2021). mHealth for pre-exposure prophylaxis adherence by young adult men who have sex with men. Mhealth.

[26] Kim HC, Pollack LM, Saberi P (2021). Study protocol: a pilot randomised waitlist-controlled trial of a dyadic mobile health intervention for black sexual-minority male couples with HIV in the USA. BMJ Open.

[27] Bauermeister J, Sullivan PS, Gravens L (2018). Reducing HIV vulnerability through a multilevel life skills intervention for adolescent men (the iREACH project): protocol for a randomized controlled trial. JMIR Res Protoc.

[28] Biello KB, Daddario SR, Hill-Rorie J (2022). Uptake and acceptability of Mychoices: results of a pilot RCT of a mobile app designed to increase HIV testing and prep uptake among young American MSM. AIDS Behav.

[29] Whiteley L, Craker L, Haubrick KK (2021). The impact of a mobile gaming intervention to increase adherence to pre-exposure prophylaxis. AIDS Behav.

[30] Songtaweesin WN, Kawichai S, Phanuphak N (2020). Youth-friendly services and a mobile phone application to promote adherence to pre-exposure prophylaxis among adolescent men who have sex with men and transgender women at-risk for HIV in Thailand: a randomized control trial. J Int AIDS Soc.

[31] Shrestha R, Altice FL, Khati A (2023). Clinic-integrated smartphone app (JomPrEP) to improve uptake of HIV testing and pre-exposure prophylaxis among men who have sex with men in Malaysia: mixed methods evaluation of usability and acceptability. JMIR Mhealth Uhealth.

[32] Schnall R, Kuhns LM, Pearson C (2022). Efficacy of Mypeeps mobile, an HIV prevention intervention using mobile technology, on reducing sexual risk among same-sex attracted adolescent males: a randomized clinical trial. JAMA Netw Open.

[33] Mustanski B, Parsons JT, Sullivan PS, Madkins K, Rosenberg E, Swann G (2018). Biomedical and behavioral outcomes of Keep It Up!: an eHealth HIV prevention program RCT. Am J Prev Med.

[34] Mitchell JT, LeGrand S, Hightow-Weidman LB (2018). Smartphone-based contingency management intervention to improve pre-exposure prophylaxis adherence: pilot trial. JMIR Mhealth Uhealth.

[35] Mitchell JT, Burns CM, Atkinson B (2022). Feasibility, acceptability, and preliminary efficacy of a gamified mobile health contingency management intervention for PrEP adherence among Black MSM. AIDS Behav.

[36] McCoy SI, Buzdugan R, Grimball R (2018). Stick To It: pilot study results of an intervention using gamification to increase HIV screening among young men who have sex with men in California. Mhealth.

[37] Mauka W, Mbotwa C, Moen K (2021). Development of a mobile health application for HIV prevention among at-risk populations in urban settings in East Africa: a participatory design approach. JMIR Form Res.

[38] Luo Q, Luo Y, Li T, Cui T (2022). An integrated online-to-offline model for HIV post-exposure prophylaxis (O2O-PEP) scale-up among men who have sex with men (MSM): protocol for developing a pilot randomized controlled trial. Front Public Health.

[39] Liu AY, Vittinghoff E, von Felten P (2019). Randomized controlled trial of a mobile health intervention to promote retention and adherence to preexposure prophylaxis among young people at risk for human immunodeficiency virus: the EPIC study. Clin Infect Dis.

[40] Liu A, Coleman K, Bojan K (2019). Developing a mobile app (LYNX) to support linkage to HIV/sexually transmitted infection testing and pre-exposure prophylaxis for young men who have sex with men: protocol for a randomized controlled trial. JMIR Res Protoc.

[41] LeGrand S, Knudtson K, Benkeser D (2018). Testing the efficacy of a social networking gamification app to improve pre-exposure prophylaxis adherence (P3: Prepared, Protected, emPowered): protocol for a randomized controlled trial. JMIR Res Protoc.

[42] Horvath KJ, Oakes JM, Rosser BRS (2013). Feasibility, acceptability and preliminary efficacy of an online peer-to-peer social support ART adherence intervention. AIDS Behav.

[43] Hightow-Weidman LB, LeGrand S, Muessig KE (2019). A randomized trial of an online risk reduction intervention for young black MSM. AIDS Behav.

[44] Hightow-Weidman L, Muessig K, Knudtson K (2018). A gamified smartphone app to support engagement in care and medication adherence for HIV-positive young men who have sex with men (AllyQuest): development and pilot study. JMIR Public Health Surveill.

[45] Dworkin MS, Lee S, Chakraborty A (2019). Feasibility, and preliminary efficacy of a theory-based relational embodied conversational agent mobile phone intervention to promote HIV medication adherence in young HIV-positive African American MSM. AIDS Educ Prev.

[46] Besoain F, Perez-Navarro A, Jacques Aviñó C, Caylà JA, Barriga NA, Garcia de Olalla P (2020). Prevention of HIV and other sexually transmitted infections by geofencing and contextualized messages with a gamified app, UBESAFE: design and creation study. JMIR Mhealth Uhealth.

[47] Andrade-Romo Z, Chavira-Razo L, Buzdugan R, Bertozzi E, Bautista-Arredondo S (2020). Hot, horny and healthy-online intervention to incentivize HIV and sexually transmitted infections (STI) testing among young Mexican MSM: a feasibility study. Mhealth.

[48] Garg PR, Uppal L, Mehra S, Mehra D (2020). Mobile health app for self-learning on HIV prevention knowledge and services among a young Indonesian key population: cohort study. JMIR Mhealth Uhealth.

[49] Laine TH, Duong N, Lindvall H, Oyelere SS, Rutberg S, Lindqvist AK (2022). A reusable multiplayer game for promoting active school transport: development study. JMIR Serious Games.

[50] DerSimonian R, Laird N (1986). Meta-analysis in clinical trials. Control Clin Trials.

[51] Higgins JPT, Thompson SG, Deeks JJ, Altman DG (2003). Measuring inconsistency in meta-analyses. BMJ.

[52] Higgins JPT, Thomas J, Chandler J (2023). Cochrane Handbook for systematic reviews of interventions. Cochrane Training.

[53] Landis JR, Koch GG (1977). The measurement of observer agreement for categorical data. Biometrics.

[54] Xu L, Shi H, Shen M (2022). The effects of mHealth-based gamification interventions on participation in physical activity: systematic review. JMIR Mhealth Uhealth.

[55] Vermeir JF, White MJ, Johnson D, Crombez G, Van Ryckeghem DML (2020). The effects of gamification on computerized cognitive training: systematic review and meta-analysis. JMIR Serious Games.

[56] HIV surveillance reports. Centers for Disease Control and Prevention.

[57] Mustanski B, Fisher CB (2016). HIV rates are increasing in gay/bisexual teens: IRB barriers to research must be resolved to bend the curve. Am J Prev Med.

[58] Drummond D, Monnier D, Tesnière A, Hadchouel A (2017). A systematic review of serious games in asthma education. Pediatr Allergy Immunol.

[59] Montagni I, Mabchour I, Tzourio C (2020). Digital gamification to enhance vaccine knowledge and uptake: scoping review. JMIR Serious Games.

[60] Simoni JM, Frick PA, Pantalone DW, Turner BJ (Nov-Dec 2003). Antiretroviral adherence interventions: a review of current literature and ongoing studies. Top HIV Med.

[61] Fisher JD, Amico KR, Fisher WA, Harman JJ (2008). The information-motivation-behavioral skills model of antiretroviral adherence and its applications. Curr HIV/AIDS Rep.

[62] Horvath KJ, Amico KR, Erickson D (2018). Thrive With Me: protocol for a randomized controlled trial to test a peer support intervention to improve antiretroviral therapy adherence among men who have sex with men. JMIR Res Protoc.

[63] Hightow-Weidman LB, Bauermeister JA (2020). Engagement in mHealth behavioral interventions for HIV prevention and care: making sense of the metrics. Mhealth.

