# Supplementary material for: mHealth-Based Gamification Interventions Among Men Who Have Sex With Men in the HIV Prevention and Care Continuum: Systematic Review and Meta-Analysis
Source: JMIR Mhealth Uhealth. 2024 Apr 15;12:e49509. doi: 10.2196/49509 (PMC11034423; doi:10.2196/49509)
Supplement: Multimedia Appendix 1 [file mhealth-v12-e49509-s001.docx]

# Appendix 2. Search strategies in each database

**Table S2-1.** Number of citations by each database

| Databases | Number |
| --- | --- |
| PubMed | 29 |
| Embase | 53 |
| Cochrane Library | 5 |
| Web of Science | 40 |
| Scopus | 184 |
| JMIR and its sister journals | 37 |
| Manual search | 1 |
| Total | 349 |

**Search strategy for PubMed (January 15, 2024)**

1. (HIV[Mesh] OR PLWH[Title/Abstract] OR HIV infect*[Title/Abstract] OR human immunodeficiency virus[Title/Abstract] OR acquired immunodeficiency[Title/Abstract] OR acquired immune deficiency[Title/Abstract] OR AIDS[Title/Abstract] OR acquired immunodeficiency syndrome[Title/Abstract] OR acquired immune deficiency syndrome[Title/Abstract] OR acquired immune-deficiency syndrome[Title/Abstract] OR acquired immuno-deficiency syndrome[Title/Abstract] OR HIV-positive[Title/Abstract] OR HIV/AIDS[Title/Abstract] OR hiv1[Title/Abstract] OR hiv2[Title/Abstract] OR HIV diagnos*[ Title/Abstract] OR serodiagnos*[Title/Abstract] OR HIV acquisition [Title/Abstract] OR acquir* HIV[Title/Abstract])
2. (Homosexuality, Male[Mesh] OR Sexual Minorities[Title/Abstract] OR Minorit*, Sexual[Title/Abstract] OR Sexual Minority[Title/Abstract] OR Gay[ Title/Abstract] OR Men Who Have Sex With Men[Title/Abstract] OR MSM[ Title/Abstract] OR Gender Minorit*[ Title/Abstract] OR Minorit*, Gender[Title/Abstract] OR Homosexual*[ Title/Abstract] OR Male Homosexuality[Title/Abstract])
3. (telemedicine[Mesh] OR telemedicine[Text Word] OR telehealth[Text Word] OR e-health[Text Word] OR m-Health[Text Word] OR eHealth[Text Word] OR mHealth[Text Word] OR ‘mobile applications’[Mesh] OR applications[Text Word] OR application[Text Word] OR app[Text Word] OR apps[Text Word] OR online[Text Word] OR mobile[Text Word] OR internet[Text Word] OR ‘web based’[Text Word] OR Smartphone[Mesh] OR ‘phone, smart’[Text Word] OR ‘smart phones’[Text Word] OR smartphones[Text Word] OR ‘smart phone’[Text Word] OR ‘phones, smart’[Text Word] OR Smartphone[Text Word] OR ‘cell phone’[Text Word] OR iphone[Text Word] OR android[Text Word] OR iOS[Text Word] OR ‘Wearable Electronic Devices’[Mesh] OR website[Text Word] OR digital*[Text Word] OR system*[Text Word] OR electronic*[Text Word] OR technolog*[Text Word] OR device[Text Word] OR framework*[Text Word] OR ‘social support’[Mesh:noexp] OR Facebook[Text Word] OR ‘networks, social’[Text Word])
4. (‘gamification’[Text Word] OR “game-based learning” [Text Word] OR gaming[Text Word] OR gamif*[Text Word] OR “game element*” [Text Word] OR game*[Text Word] OR “game mechanic*” [Text Word] OR gameful*[Text Word] OR “game design element*” [Text Word] OR “game-design element*” [Text Word] OR “game interface element*” [Text Word] OR “game feature” [Text Word] OR “game-like element*” [Text Word] OR “videogame element*” [Text Word])
5. 1 AND 2 AND 3 AND 4
6. Filters: English

**Search strategy for Embase (January 15, 2024)**

1. HIV/exp OR PLWH:ti,ab OR “HIV infect*”:ti,ab OR “human immunodeficiency virus”:ti,ab OR “acquired immunodeficiency”:ti,ab OR “acquired immune deficiency”:ti,ab OR AIDS:ti,ab OR “acquired immunodeficiency syndrome”:ti,ab OR “acquired immune deficiency syndrome”:ti,ab OR “acquired immune-deficiency syndrome”:ti,ab OR “acquired immuno-deficiency syndrome”:ti,ab OR "HIV-positive":ti,ab OR "HIV/AIDS":ti,ab OR hiv1:ti,ab OR hiv2:ti,ab OR "HIV diagnos*":ti,ab OR serodiagnos*:ti,ab OR "HIV acquisition":ti,ab OR “acquir* HIV":ti,ab
2. “Homosexuality, Male”/exp OR “Sexual Minorities”:ti,ab OR “Minorit*, Sexual”:ti,ab OR “Sexual Minority”:ti,ab OR Gay*:ti,ab OR “Men Who Have Sex with Men”:ti,ab OR MSM*:ti,ab OR “Gender Minorit*”:ti,ab OR “Minorit*, Gnder”:ti,ab OR Homosexual*:ti,ab OR “Male Homosexuality”:ti,ab
3. Telemedicine/exp OR telehealth/exp OR e-health/exp OR m-Health/exp OR eHealth/exp OR mHealth/exp OR applications:ti,ab OR application/exp OR app/exp OR apps:ti,ab OR online/exp OR mobile OR internet/exp OR “web based” OR ‘Smartphone’/exp OR ‘phone, smart’/exp OR ‘smart phones’/exp OR ‘smartphones’/exp OR ‘smart phone’/exp OR ‘phones, smart’/exp OR ‘Smartphone’/exp OR ‘cell phone’/exp OR ‘iphone’/exp OR android/exp OR iOS/exp OR ‘Wearable Electronic Devices’:ti,ab OR website/exp OR digital*/exp OR system*/exp OR electronic*/exp OR technolog*/exp OR device/exp OR framework*/exp OR ‘social support’:ti,ab OR ‘Facebook’/exp OR ‘networks, social’/exp
4. “gamification”/exp OR “game-based learning”/exp OR gaming/exp OR gamif*/exp OR “game element*”/exp OR game*/exp OR “game mechanic*”/exp OR gameful*/exp OR “game design element*”/exp OR “game-design element*”/exp OR “game interface element*”/exp OR “game feature”/exp OR “game-like element*”/exp OR “videogame element*”/exp
5. 1 AND 2 AND 3 AND 4
6. Filters: English

**Search strategy for the Cochrane Library (January 15, 2024)**

1. [mh HIV] OR PLWH:ti,ab OR “HIV NEXT infect*”:ti,ab OR “human immunodeficiency virus”:ti,ab OR “acquired immunodeficiency”:ti,ab OR “acquired immune deficiency”:ti,ab OR AIDS:ti,ab OR “acquired immunodeficiency syndrome”:ti,ab OR “acquired immune deficiency syndrome”:ti,ab OR “acquired immune-deficiency syndrome”:ti,ab OR “acquired immuno-deficiency syndrome”:ti,ab OR "HIV-positive":ti,ab OR "HIV/AIDS":ti,ab OR “hiv1”:ti,ab OR “hiv2”:ti,ab OR "HIV NEXT diagnos*":ti,ab OR serodiagnos*:ti,ab OR "HIV acquisition":ti,ab OR "acquir* NEXT HIV":ti,ab
2. [mh “Sexual and Gender Minorities”] OR [mh “homosexuality, Male”] OR LGBTQ:ti,ab OR “minority, Sexual”:ti,ab OR bisexual*:ti,ab OR Gay:ti,ab OR “men who have sex with men”:ti,ab OR Homosexual:ti,ab OR “gender minority”:ti,ab “male homosexuality”:ti,ab
3. "telemedicine" OR "telemedicine" OR "telehealth" OR e-health OR m-Health OR eHealth OR mHealth OR "mobile applications":ti,ab OR "applications" OR "application" OR app OR apps OR "online" OR "mobile" OR "internet" OR “web based” OR "Smartphone" OR "phone, smart" OR "smart phones" OR "smartphones" OR "smart phone" OR "phones, smart" OR "Smartphone" OR “cell phone” OR iphone OR android OR iOS OR "Wearable Electronic Devices":ti,ab OR website OR digital* OR system* OR electronic* OR technolog* OR device OR framework* OR "social support":ti,ab OR "Facebook" OR "networks, social"
4. gamification OR game OR "game-based learning" OR gamif* OR "game element*" OR game* OR "game mechanic*" OR gameful* OR "game design element*" OR "game interface element*" OR "game feature" OR "game-like element*" OR "videogame element*"
5. Filters: English

**Search strategy for the Web of Science (January 15, 2024)**

1. TS= (HIV OR PLWH OR “HIV infect*” OR “human immunodeficiency virus” OR “acquired immunodeficiency” OR “acquired immune deficiency” OR AIDS OR “acquired immunodeficiency syndrome” OR “acquired immune deficiency syndrome” OR “acquired immune-deficiency syndrome” OR “acquired immuno-deficiency syndrome” OR “HIV-positive” OR HIV/AIDS OR hiv1 OR hiv2 OR “HIV diagnos*” OR serodiagnos* OR “HIV acquisition” OR “acquir* HIV”)
2. TS= (“Homosexuality, Male” OR “Sexual Minorities” OR “Minorit*, Sexual” OR “Sexual Minority” OR Gay OR “Men who Have Sex With Men” OR MSM OR “Gender Minorit*” OR “Minorit*, Gender” OR Homosexual* OR “Male Homosexuality”)
3. TS= ("telemedicine" OR "telehealth" OR e-health OR m-Health OR eHealth OR mHealth OR "mobile applications" OR "applications" OR "application" OR app OR apps OR "online" OR "mobile" OR "internet" OR “web based” OR "Smartphone" OR "phone, smart" OR "smart phones" OR "smartphones" OR "smart phone" OR "phones, smart" OR "Smartphone" OR “cell phone” OR iphone OR android OR iOS OR "Wearable Electronic Devices" OR website OR digital* OR system* OR electronic* OR technolog* OR device OR framework* OR "social support" OR "facebook" OR "networks, social")
4. TS= (“gamification” OR “game-based learning” OR gaming OR gamif* OR “game element*” OR game* OR “game mechanic*” OR gameful* OR “game design element*” OR “game-design element*” OR “game interface element*” OR “game feature” OR “game-like element*” OR “videogame element*”)
5. 1 AND 2 AND 3 AND 4
6. Filters: English

**Search strategy for Scopus (January 15, 2024)**

1. TITLE-ABS-KEY (HIV OR PLWH OR “HIV infect*” OR “human immunodeficiency virus” OR “acquired immunodeficiency” OR “acquired immune deficiency” OR AIDS OR “acquired immunodeficiency syndrome” OR “acquired immune deficiency syndrome” OR “HIV-positive” OR “HIV/AIDS” OR hiv1 OR hiv2 OR “HIV diagnos*” OR serodiagnos* OR “HIV acquisition” OR “acquir* HIV”)
2. TITLE-ABS-KEY (“Sexual Minorities” OR Minorit*, Sexual OR “Sexual Minority” OR Gay OR “Men Who Have Sex With Men” OR MSM OR ”Gender Minorit*” OR Homosexual* OR “Male Homosexuality”)
3. ALL (telemedicine OR telehealth OR e-health OR eHealth OR mHealth OR "mobile applications" OR applications OR application OR app OR apps OR internet OR “web based” OR Smartphone OR “cell phone” OR iphone OR android OR iOS OR "Wearable Electronic Devices" OR website OR digital* OR system* OR electronic* OR technolog* OR device OR framework* OR "social support" OR Facebook OR "networks, social")
4. ALL (gamification OR “game-based learning” OR gaming OR gamif* OR “game element*” OR game* OR “game mechanic*” OR gameful* OR “game design element*” OR “game interface element*” OR “game feature” OR “game-like element*” OR “videogame element*”)
5. 1 AND 2 AND 3 AND 4
6. Filters: English

**Search strategy for the Journal of Medical Internet Research (January 15, 2024)**

To identify relevant articles, we used the search tool on the JMIR (<https://www.jmir.org/search>) website to search for studies containing the following terms:

1. Title/Abstract/Keyword: HIV

AND

1. Title/Abstract/Keyword: men who have sex with men

AND

1. Title/Abstract/Keyword: telemedicine OR mHealth OR eHealth

AND

1. Title/Abstract/Keyword: gamification
