# Supplementary material for: mHealth-Based Gamification Interventions Among Men Who Have Sex With Men in the HIV Prevention and Care Continuum: Systematic Review and Meta-Analysis
Source: JMIR Mhealth Uhealth. 2024 Apr 15;12:e49509. doi: 10.2196/49509 (PMC11034423; doi:10.2196/49509)
Supplement: Multimedia Appendix 2 [file mhealth-v12-e49509-s002.docx]

# Appendix 3. Summary of studies included in the review

**Table S3-1.** Study information and participant characteristics

| Study | Country | Study design | Study setting | Study population | Recruitment method | Recruitment period | Sample size | HIV status | age | Outcome | Follow-up times | Retention |
| --- | --- | --- | --- | --- | --- | --- | --- | --- | --- | --- | --- | --- |
| Wray 2023 | USA | RCT 1:1 | EHE jurisdictions | Heavy drinking MSM | online | 2021.6-2022.2 | 73 | HIV- | 21-59 | PrEP adherence | 6 months | 1-m: 97%  3-m: 93%  6-m: 75% |
| Weitzman 2021 | USA | Pre-post | Boston | MSM | Offline+  online | Not mentioned | 54 | HIV- | 25.6  (2.27) | PrEP adherence | 6-weeks | 6-w: 100% |
| Kim 2021 | USA | protocol | High HIV infection area in US | Black MSM | Offline+ online | NR |  | HIV+ |  | ART adherence |  |  |
| Bauermeister 2018 | USA | Protocol | Four regions | Adolescent MSM | Offline+ online | NR | 600 | HIV- | 13-18 years | HIV testing | 15 months | NA |
| Biello 2022 | USA | 2:1 RCT | Three regions | Young MSM | Offline+ online | 2018.11-2019.4 | 60 | HIV-/unknown | 21.5 (2.1) | HIV testing  PrEP initiation | 6 months | 6-m: 90% |
| Whiteley 2021 | USA | RCT | PrEP clinics | MSM | Offline | 2017-2019 | 81 | HIV- | 25.1 (4.2) | PrEP adherence | 24 weeks | 12-w: 63/81=77.8%  24-w: 55/81=67.9% |
| Shrestha 2023 | Malaysia | Usability test | The Great Kuala Lumpur region | MSM | Offline+ online | 3-4/2022 | 50 | Self-reporting HIV- | 27.9 (5.3) | Uptake of HIV testing and PrEP | 30 days | 100% |
| Mustanski 2018 | USA | RCT 2 | Atlanta  Chicago  NY | MSM | Offline+  online | 5/2013- 12/2015 | 901 | Rapid test HIV- | 18-24: 477  25-29: 423 | STI  ACS | 12 months | 3-m: 86.2%  6-m: 81.2%  12-m: 85.6% |
| Hightow-Weidman 2019 | USA | RCT 2 | North California | MSM | Offline+ online | 11/2013-5/2015 | 474 | HIV+(42%)  HIV-(58%) | 24.33 (3.22) | Number of CAI | 12 months | 3-m:85%  6-m:80%  12-m:78% |
| Mitchell 2022 | USA | Pre-post | Community, clinics. | YBMSM | Offline | NR | 15 | HIV- | 25.60 (2.10) | PrEP adherence | 8 weeks | 15/16=93.8% |
| Mitchell 2018 | USA | Pre-post | Community | YMSM | Offline | NR | 10 | HIV- | 24.10 (2.38) | PrEP adherence | 4 weeks | 10/12=83.3% |
| McCoy 2018 | USA | Historical control trial | 2 clinics in California.  O2O | YMSM | Offline+ online | 10/2016-6/2017 | 166 | NR | 23 | Repeat HIV testing | 6-m | 164/166=98.8% |
| Mauka 2021 | Tanzania | Design and feasibility test | Dar es salaam in Tanzania | MSM | Offline | NR | 20 | HIV- | 27 (21-44) | PrEP adherence | 2-w | 19/20=95.0% |
| Liu 2019 | USA | RCT 2:1  protocol | Chicago  Tampa | YMSM | Online+ offline | NR | 60 | HIV- |  | HIV/STI test and PrEP uptake | 24-w | NA |
| Hightow-Weidman 2021 | USA | RCT1:1 | Clinics and AIDS service organizations | YMSM | Offline | 10/20215-9/2016 | 146 | HIV+ | 21.3 (2) | VL suppression | 39-w | 13-w: 78%  26-w: 76%  39-w:66% |
| LeGrand 2018 | USA | RCT1:1:1  protocol | 6 US cities | YMSM | Online+ offline | NR | 240 | HIV- | NA | PrEP adherence | 6-m | NA |
| Schnall 2022 | USA | RCT1:1 | 4 US cities | Adolescent MSM | Online+ offline | 7/2018-4/2020 | 763 | Report HIV- OR unknown | 16.2 (1.4) | Condomless anal sex | 9-m | 3-m: 86.4%  6-m: 83.4%  9-m: 81.8% |
| Horvath 2013 | USA | RCT1:1 | New York | MSM | Online+ offline | 2-4/2011 | 123 | HIV+ | 42.7 (10) | ART adherence | 3-m | 90.2% |
| HightowWeidman 2018 | USA | Feasibility test | Chicago | YMSM | NR | 10/2016-1/2017 | 20 | HIV+ | 21.8 (1.55) | ART adherence | 1-m | 17/20=85% |
| Garg 2020 | Indonesian | Pre-post | Indonesia | MSM | Offline | 8/2017 | 50 | NR | 22.6 (3.3) | High risk behavior | 3-m | 49/50=98% |
| Dworkin 2019 | USA | Pre-post | Chicago | MSM | Offline | 1-12/2017 | 43 | HIV+ | 29 (18-34) | ART adherence | 3-m | 32/43=74% |
| Besoain 2020 | Spain | Feasibility test | Spain | MSM | Offline | NR | 5 | HIV- | 36.2 (7.66) | High risk behavior | 2-w | 4/5=80% |
| Andrade-Romo  2020 | Mexico | Feasibility test | Mexico | MSM | Offline | 10-11/2015 | 61 | HIV- | 25.0 | HIV test | NR | NR |
| Songtaweesin 2020 | Thailand | RC1:1 | Bangkok | MSM (74%)  TG (26%) | Offline+ online | 3/2018-6/2019 | 200 | HIV- | 18 (17-19) | PrEP adherence | 6-m | 73% |
| Liu 2019 | USA | RCT2:1 | Chicago | YMSM (96%)  TG (4%) | Offline +online | 4/2015-3/2016 | 121 | HIV- | 24.2 (18-29) | PrEp adherence | 12-m | 89.3% |
| Luo 2022 | China | RCT protocol | Shandong | MSM | Offline+ online | NR | 50 | HIV- | NA | PEP initiation | 6-m | NA |

**Table S3-2.** The gamification framework---Octalysis

| Core drives | Explanation | Examples |
| --- | --- | --- |
| Epic meaning & calling | One is doing something greater than himself or he is chosen to do something | Narratives, Humanity Hero, Elitism, Beginner’s luck, Free lunch. |
| Development & accomplishment | People are driven by a sense of growth and a need to accomplish a targeted goal | Progress Bars, The rockstar effect, Achievement symbols, Status points, Leaderboards. |
| Empowerment creativity & feedback |  | Boosters, Milestone unlock, Point picker, Plant picker |
| Ownership & possession | Motivation that is driven by feelings of owning something, and consequently the desire to improve, protect, and obtain more. | Build-From-Scratch, Collection sets, Exchangeable points, Monitor attachment, The Alfred effect. |
| Social influence & relatedness | It involves activities inspired by what other people think, do, or say. | Mentorship, Brag buttons, Trophy shelves, Group quests, Social treasures, Social prods, Conformity anchor, Water coolers. |
| Scarcity & impatience | Drive that motivates people simply because they are either unable to have something immediately, or because there is great difficulty in obtaining it. | Dangling, Anchored Juxtaposition, Magnetic caps, Appointment dynamics, Torture breaks, Evolved UI. |
| Unpredictability & curiosity | The main force behind infatuation with experiences that are uncertain and involve chance. | Glowing choice, Mystery boxes, Random rewards, Easter eggs, Lottery |
| Loss & avoidance | It motivates through the fear of losing something or having undesirable events transpire. | Rightful heritage, Evanescent opportunities, Countdown timers |

Reference: Yu-Kai Chou. Actionable Gamification: Beyond Points, Badges, and Leaderboards. 2014.

**Table S3-3**. Intervention characteristics

| Studies | Controls | Interventions | Developer | Intervention content | Intervention module | Modality | Core drives | Game components | Theories |
| --- | --- | --- | --- | --- | --- | --- | --- | --- | --- |
| Wray 2023 | Attention-matched: educational videos on sleep hygiene and diet. | *Game plan for PrEP* | Y | Providing feedback about HIV infection risk and STIs | 12 Onboarding; About you;Your sex life; Your risk; About PrEP; Your drinking habits; Alcohol, sex and PrEP; Alcohol use profile and norms; Pros and cons exercise; Your game plan; Planting a seed; Local resources | Laptop/desktop computer | 1) Development & accomplishment  2)Empowerment creativity & feedback | Challenges,  Tailored message | MI |
| Weitzman 2021 | NA | *Dot* | Y | Personalized pill reminders with positive psychology-based texts | 3 security & privacy; Technology advantages; Assistance | Smartphone | 1)Empowerment creativity & feedback | Emoji enhancement | N |
| Kim 2021 | Waitlist control | *LetSync* | Y | Action plan and coordination to solve problems in ART adherence | 1 My action plan | iOS/Android app | 1) Development & accomplishment  2)Empowerment creativity & feedback  3) Social influence & relatedness | Challenges  Progress  Partner coordination | Dyadic HIV Care Engagement |
| Bauermeister 2018 | Local resource information | *iREACH* | NR | Life skills intervention | 4 Essentials; My goals; Locator; Sessions | app | 1) Development & accomplishment  2)Empowerment creativity & feedback  3) Social influence & relatedness  4) Ownership & possession | Challenges  Progress  Peer mentor  badges | NR |
| Biello 2022 | Standard of care | *MyChoices* | Y |  | 5 Sexual health information; HIV testing plans’ Reminders; Service maps; Free test kits, condoms and lube | app | 1) Development & accomplishment | Challenges | Social cognitive theory |
| Whiteley 2021 | Non-PrEP non-IBM-informed game | *Viral Combat* | Y | Participant fight off HIV and keep it from entering the body. | 3 Games fighting virus on the surface of the skin, in the arterial system, in the penile and anal canals; HIV-related educational material; Adherence message. | iPhone app | 1) Ownership & possession  2) Development & accomplishment  3)Empowerment creativity & feedback | Points,  Challenges,  Tailored messages | IMB |
| Shrestha 2023 | NA | JomPrEP | Y | Offering a range of HIV prevention and other support services | 13 Home page; HIVST; PrEP Express; orders; labs; appointments; mental health; Medmanager;MoodTracker;Resources; News; Reward points; clinic dashboard | Android/iOS app | 1) Ownership & possession | Reward points | SCT |
| Mustanski 2018 | Existing and generally available online information of HIV prevention | KIU! | Y | Using various types of content (video, games, animation) to increase HIV knowledge, motivate safer behavior, and self-efficacy for HIV prevention. | 7 Healthy and whole person; Hooking up online; The club game; Dating; A serious relationship; Setting risk reduction goals; Sex in the city. | Tablets/computers | 1) Social influence & relatedness | Mentorship | IMB |
| HightowWeidman 2019 | 110 articles focused on HIV and STI prevention | HMP | NR | To increase safer sex behaviors among HIV+ and HIV- BMSM | 4 The Forum; Getting Real; Ask Dr. W; Decision support tools | Internet/ Mobile phone | 1) Ownership & possession  2)Empowerment creativity & feedback | Points  Online doctor responding questions within 72h | IBM |
| Mitchell 2022 | NA | mSMART | N | Including six components to target PrEP adherence | 6 Medication aide; SMART desk; Adherence strategies; Coping strategies; Prescription and doses; Treatment progress. | Android/iOS app | 1) Ownership & possession  2) Unpredictability & curiosity | Bounus draws for: non-monetary reinforcer (65.5% chance), $1 (26.7% chance, $20 (0.2% chance) | IMB |
| Mitchell 2018 | NA | mSMART | N | Including components to target PrEP adherence | 6 Medication aide; SMART desk; Adherence strategies; Coping strategies; Prescription and doses; Treatment progress. | Android/iOS app | 1) Ownership & possession | Money | IMB |
| McCoy 2018 | NA | Stick-to-it | Y | To increase repeat HIV/STI screening | 3 Online enrollment; web-based activities; in-person activities. | NR | 1) Ownership & possession  2) Unpredictability & curiosity | Points  Gumball draws | N |
| Mauka 2021 | NA | Jichunge | Y | To promote PrEP adherence | 10 Levels and points; Gamification; Drug registration; Medication time reminder; Notification; Communication with peers; Communication with a health care provider; Discussion forum; Educational materials; Jichunge quiz. | app | 1) Ownership & possession  2) Development & accomplishment  3) Social influence & relatedness | Points;  Different levels;  Discussion forum. | ISR |
| Liu 2019 | Provision of information on HIV test and prevention | LYNX | Y | To promote HIV/STI test and PrEP uptake | 5 onboarding page; Diary; Testing; PrEP; Chat. | Android/iPhone app | 1) Ownership & possession | Badges | IMB |
| HightowWeidman 2021 | Receive a weekly brief informational article for 26 weeks | Epic Allies | Y | To promote ART adherence. | 6 Medication reminders; User survey on ART; User profile; Health center; Interact with allies; The daily dose. | Android/iPhone app | 1) Ownership & possession  2) Development & accomplishment  3) Social influence & relatedness | Badges  Game level  Allegiance | IMB |
| LeGrand 2018 | Standard of care | G1: P3  G2: P3+ | Y | To promote PrEP adherence | 7 Profile page; daily discussion; medication tracking and adherence support; brain builders, brain builders; knowledge center; character-based narratives | Android/iPhone app | 1) Ownership & possession  2) Development & accomplishment  3) Social influence & relatedness | Bank count, in-game currency  Game level  Character-based narratives | SCT; narrative communication; persuasive technology |
| Schnall 2022 | Delayed intervention | MyPEEPS | Y | Condomless anal sex acts | 4 Introduction; #realtalk; P woke up like this; Making tough situations LITuations. | App | 1) Social influence & relatedness  2) Development & accomplishment | Stories of 4 “peeps”  BottomLine | Social learning theory |
| Horvath 2013 | Weekly email with information similar to a newsletter | Thrive with Me (TWM) | Y | ART adherence | 3 private social networking feature; tailored HIV/ART adherence information; medication reminders, self-monitoring, reflection. | Website | 1) Ownership & possession  2) Social influence & relatedness | Points, badges  Asynchronous peer-to peer interaction | IMB |
| HightowWeidman 2018 |  | AllyQuest | Y | ART adherence | 6 profile page; daily discussion; medication tracker; brain builders; knowledge center; character-based narratives | App | 1) Ownership & possession  2) Development & accomplishment  3) Social influence & relatedness | Virtual currency  Level-up  discussion | SCT,  storyline |
| Garg 2020 | No control | RUMAH SELA | Y | High risk behavior, HIV testing | 7 Number of downloads per province; games; map of facilities; ask a question; health facility resources; stigma score; take the test. | Android app | NR | NR | Principle of self-learning |
| Dworkin 2019 | No control | My Personal Health Guide | Y | ART adherence | 3 Let me explain, medication manager, settings. | Android app | 1) Social influence & relatedness | An embodied conversational agent | IMB |
| Besoain 2020 | No control | UBESAFE | Y | High risk behavior | 5 app patrol; map patrol; managing health messages; gamification scoreboard; URL patrol. | Android app | 1) Ownership & possession  2) Social influence & relatedness | Points of experience, medals, and ranking.  Share information with others. | NR |
| Andrade-Romo  2020 | No control | NR | Y | HIV test | 4 gamification; information; linkage to prevention and testing services; Game | Internet/app | 1) Development & accomplishment  2) Ownership & possession | Leaderboard  Points, badges | SCT |
| Songtaweesin 2020 | Young friendly service | Project Raincoat | Y | PrEP adherence | 3 HIV risk assessment; data input; reminder. | Android app | 1) Ownership & possession | Points | IMB |
| Liu 2019 | Standard of care | PrEPmate | Y | PrEP adherence | PrEP basics, videos and testimonials of peers taking PrEP, support forum. | Internet | 1) Social influence & relatedness | Videos of peers,  Online supporting forum. | IMB |
| Luo 2022 | Routine PEP-related care | O2O-PEP | Y | To increase PEP initiation | 4 a discussion forum; three games; e-counseling; online booking system. | App | 1) Ownership & possession  2) Unpredictability & curiosity  3) Social influence & relatedness | Points  Different cards  Discussion forum, e-counseling | Levesque framework |

Controls: nonactive

Developers: whether including intervention target participants: yes (Y) VS. no (N).

MI: Motivational Interviewing. IMB: Information, Motivation, Behavioral skill. SCT: Social Cognitive Theory； IBM: integrated behavioral model; ISR: information system research framework; SCT: social cognitive theory;

Table S3-4. Outcome characteristics

| Study | Primary outcome | | | | | | | | | | | |  | | Secondary outcome | | | | | | | | | |
| --- | --- | --- | --- | --- | --- | --- | --- | --- | --- | --- | --- | --- | --- | --- | --- | --- | --- | --- | --- | --- | --- | --- | --- | --- |
|  | Name | Measures | Definition | time | N1 | | Total1 | | N0 | Total0 | | Name | | Measures | | Definition | Time | N1 | Total1 | | N0 | | Total0 | |
| Wray 2023 | PrEP adherence | Self-report | >=4 days per week in past 30 days | 6-m | 34 | | 37 | | 33 | 36 | |  | |  | |  |  |  |  | |  | |  | |
| Weitzman 2021 | PrEP adherence | Self-report | Reported “no” to all the 3 questions | 6-w | 39 | | 54 | | 21 | 54 | |  | |  | |  |  |  |  | |  | |  | |
| Biello 2022 | HIV testing | Self-report | Self-reporting HIV test in the follow-up | 3-m | 19 | | 40 | | 10 | 20 | | CAS | | Self-report | | Engagement in condomless sex in the prior 3 months with male non-primary partners | 3-m | 14 | 40 | | 8 | | 20 | |
|  |  |  |  | 6-m | 27 | | 40 | | 13 | 20 | |  | |  | |  | 6-m | 15 | 40 | | 7 | | 20 | |
|  | PrEP initiation | Self-report | Self-reporting PrEP uptake in the follow-up | 3-m | 0 | | 40 | | 2 | 20 | |  | |  | |  |  |  |  | |  | |  | |
|  |  |  |  | 6-m | 3 | | 40 | | 3 | 29 | |  | |  | |  |  |  |  | |  | |  | |
| Whiteley 2021 | ARV levels | Dried blood spots | Four or more days per week was considered optimal dosing | 6-m | 15 | | 27 | | 7 | 28 | |  | |  | |  |  |  |  | |  | |  | |
|  |  |  |  | 3-m | 18 | | 31 | | 14 | 32 | |  | |  | |  |  |  |  | |  | |  | |
| Shrestha 2023 | Acceptability | SUS | >50 (out of 100) indicating the app is acceptable | 30-d | 73.8 (10.1) | | NA | | NA | NA | | HIV test | | App data | | Ordering HIVST kit using the app | 30-d | 42 | 50 | | NA | | NA | |
|  | Usability | App analytics | Number of log-ins, session duration, pages visited, frequency and duration of use of app components | 30-d | 8 (5) visits; 28 (38.9) minutes per session; | | NA | | NA | NA | | PrEP uptake | | App data | | Getting on PrEP using the app | 30-d | 46 | 50 | | NA | | NA | |
| Mustanski 2018 | Incident STI | laboratory | Testing positive for urethral or rectal GC/CT at 12-month follow-up | 12-m | 31 | | 359 | | 54 | 374 | | Number of CAS acts | | Self-reporting | | - | - |  |  | |  | |  | |
|  | CAS | Self-report | CAS with casual partners in the prior 3 months | 3-m | 137 | | 370 | | 172 | 392 | | Number of CAS partners | | Self-reporting | | - | - |  |  | |  | |  | |
| Hightow-Weidman 2019 | CAI | Self-report | The number of acts of receptive or insertive condomless anal sex with a male partner | 3-m | 89 | | 238 | | 129 | 236 | | Serodiscordant CAI | | Self-report | | The number of CAI where the partner was reported as having different HIV status | 3-m | 14 | 238 | | 10 | | 236 | |
|  |  |  |  | 6-m | 21 | | 238 | | 15 | 236 | |  | |  | |  | 6-m | 68 | 238 | | 43 | | 236 | |
|  |  |  |  | 12-m | 23 | | 238 | | 21 | 236 | |  | |  | |  | 12m | 61 | 238 | | 49 | | 236 | |
| Mitchell 2022 | PrEP adherence | Blood draw: 0 (no dose of drug) to 5 (good adherence) | 4 and 5 is considered adherence of PrEP is efficacious | 8-w |  | |  | |  |  | | feasibility | | Study attrition; Function issue; engagement in SMART; | | NA | 8-w | No study attrition;  9/15 reported function issue;  82% of all participants daily mSMART use. | | | | | | |
|  |  |  |  |  |  | |  | |  |  | | Acceptability | | SUS (0-100) | | >68 | 8w | 73% (11/15) had scores in the acceptable range. Average score of SUS 72.33 (15.82). | | | | | | |
| Mitchell 2018 | PrEP adherence | Blood draw: 0 (no dose of drug) to 5 (good adherence) | 4 and 5 is considered adherence of PrEP is efficacious | 4-w | 30% improved PrEP adherence score  70% did not changed the adherence score. | | | | | | | Feasibility | | Study attrition; mSMART compatibility incidents; daily engagement with MSMART; the number of prompts. | |  | 4-w | No study attrition; no function incompatibility; | | | | | | |
|  |  |  |  |  |  | |  | |  |  | | Acceptability | | 5 individual questions (1-4);  SUS | | >68 | 4-w | Q1: 2.8 (0.63); Q2: 3.5 (0.53); Q3: 2.7 (0.82); Q4: 2.8 (0.79); Q5: 1.2 (0.42).  SUS: 68.25 (15.1), 60% acceptable. | | | | | | |
| McCoy 2018 | Acceptability | Completion of registration, onboarding, subsequent quiz, redemption of points. | Intervention engagement;  Qualitative review | Enrollment to 6-m | Participants saw value in the program in general but differed about whether it was individually motivating.  56% completed setting the testing countdown timer and answering the quiz.  19% completed ≥1 online activity.  Only 27% of those recruited offline and 5% recruited online visited the offline clinics for reward. | | | | | | | Repeat HIV screening | | Antibody test, if non-reactive, further acute and recent infection test. | | Received >=2 HIV tests over 6 months of follow-up. | 6-m | 15 | | 31 | | 157 | | 517 |
| Mauka 2021 | Usability | App web-based server. | User statistics of various app features | 2-w | Average 1/9 features were used per day, with highest in “communication with a health care provider” and “educational material” | | | | | | |  | |  | |  |  |  | |  | |  | |  |
| Hightow-Weidman 2021 | VL suppression |  | VL below the lower limit of detection. | 13-w | 36 | 59 | | 30 | | | 55 | Engagement in care | | Self-report | | Attend at least 1 HIV health care visit in the prior 3-m | 13-w | 49 | | 61 | | 47 | | 59 |
|  |  |  |  |  |  |  |  |  |  |  |  |  |  |  |  |  | 26-w | 44 | | 60 | | 40 | | 51 |
|  |  |  |  |  |  |  |  |  |  |  |  |  |  |  |  |  | 39-w | 41 | | 50 | | 36 | | 46 |
|  |  |  |  | 26-w | 39 | 62 | | 36 | | | 49 | ART uptake | | Self-report | | Not report on ART at baseline but taking ART at follow-up | 13-w | 55 | | 61 | | 59 | | 59 |
|  |  |  |  |  |  |  |  |  |  |  |  |  |  |  |  |  | 26-w | 5 | | 60 | | 3 | | 51 |
|  |  |  |  |  |  |  |  |  |  |  |  |  |  |  |  |  | 39-w | 10 | | 50 | | 2 | | 46 |
|  |  |  |  | 39-w | 36 | 57 | | 26 | | | 40 | ART adherence | | Self-report | | ≥90% adherence to ART in past 7-d | 13-w | 35 | | 61 | | 39 | | 59 |
|  |  |  |  |  |  |  |  |  |  |  |  |  |  |  |  |  | 26-w | 36 | | 60 | | 20 | | 51 |
|  |  |  |  |  |  |  |  |  |  |  |  |  |  |  |  |  | 39-w | 29 | | 50 | | 26 | | 46 |
| Schnall 2022 | CAS | Self-report | The number of recent CAS acts prior 3 months | 3-m | 382 | 0.88 (0.16) | | 381 | | | 1.1 (0.17) | Satisfaction with MyPEEPS | | Self-report | | 8-item Client Satisfaction Questionnaire | 3-m | 623 | | 97.1% rate MyPEEPS good or excellent;  93.9% recommend it to friends. | | | | |
|  |  |  |  | 6-m | 382 | 0.94 (0.17) | | 381 | | | 1.11 (0.17) |  | |  | |  |  |  | |  | |  | |  |
|  |  |  |  | 9-m | 382 | 1.39 (0.24) | | 381 | | | 1.22 (0.2) |  | |  | |  |  |  | |  | |  | |  |
| Horvath 2013 | ART adherence | Self-report the % of time ART was correctly taken as prescribed in past 30 days. | ≥90%  <90% | 3-m | 43 | 66 | | 37 | | | 57 |  | |  | |  |  |  | |  | |  | |  |
| Hightow  Weidman 2018 | Feasibility | In-app analytics |  | 1-m | 1) mean total time of app use: 158.4 (14.1) min  2) app usage days: mean 21.2 d  3) | | | | | | | ART adherence | | Self-report | | Ability to reliably take medication | 1-m |  | | | | | | |
|  | Acceptability | SUS/CSQ-8 |  | 1-m | CSQ-8: 27.8 (5.9) | | | | | | |  | |  | |  |  |  | |  | |  | |  |
| Garg 2020 | Sexual risk behavior (MSM) | Self-report | Not using condom at last sexual intercourse | 3-m | 11 | 49 | | 9 | | | 49 |  | |  | |  |  |  | |  | |  | |  |
|  | Sexual risk behavior (TG) | Self-report | Not using condom at last sexual intercourse | 3-m | 9 | 49 | | 6 | | | 49 |  | |  | |  |  |  | |  | |  | |  |
| Dworkin 2019 | ART adherence | Self-report | Pill count >80% | 3-m | 16 | 26 | | 23 | | | 26 | Acceptability | | Self-report | | The extent of app use; which functions used more; to what extent value each function; |  |  | |  | |  | |  |
| Songta-weesin 2020 | PrEP adherence | DBS sample | TFV-DP>=700 fmol/punch  >=4 doses/w | 3-m | 44 | 81 | | 40 | | | 79 | Rate of HIV infection | | Laboratory test | |  | 3-m |  | |  | |  | |  |
|  |  |  |  | 6-m | 36 | 73 | | 30 | | | 68 |  | |  | |  | 6-m |  | |  | |  | |  |
| Liu 2019 | PrEP adherence | DBS sample | TFV-DP>=700 fmol/punch  >=4 doses/w | 1-m | 73 | 81 | | 31 | | | 40 |  | |  | |  |  |  | |  | |  | |  |
|  |  |  |  | 3-m | 62 | 81 | | 26 | | | 40 |  | |  | |  |  |  | |  | |  | |  |
|  |  |  |  | 6-m | 54 | 81 | | 18 | | | 40 |  | |  | |  |  |  | |  | |  | |  |
|  |  |  |  | 12-m | 45 | 81 | | 16 | | | 40 |  | |  | |  |  |  | |  | |  | |  |

CASI: computer-assisted self-interview; EP: electronic picture; MR: medical record confirmation; CAS: condomless anal sex;
