# Supplementary material for: mHealth-Based Gamification Interventions Among Men Who Have Sex With Men in the HIV Prevention and Care Continuum: Systematic Review and Meta-Analysis
Source: JMIR Mhealth Uhealth. 2024 Apr 15;12:e49509. doi: 10.2196/49509 (PMC11034423; doi:10.2196/49509)
Supplement: Multimedia Appendix 3 [file mhealth-v12-e49509-s003.docx]

# Appendix 4. Study quality assessment

**Table S4-1**. Risk of bias assessment for RCTs with RoB2

| Study | D1 | D2 | D3 | D4 | D5 | Overall |
| --- | --- | --- | --- | --- | --- | --- |
| Whiteley 2021 | Low | Some concerns | High | Low | Low | High |
| Mustanski 2018 | Low | Low | Low | Low | Low | Low |
| Hightow-Weidman 2019 | Some concerns | Some concerns | High | Low | Low | High |
| Hightow-Weidman 2021 | Low | Some concerns | Low | Low | Low | Some concerns |
| Schnall 2022 | Low | Low | Low | Low | Low | Low |
| Horvath 2013 | Low | Low | Low | High | Low | High |
| Songtaweensin 2020 | Some concerns | Low | Some concerns | High | Low | High |
| Liu 2019 | Low | Low | Low | Low | Low | Low |
| Wray 2023 | Low | High | Low | High | Low | High |
| Beillo 2022 | Low | Some concerns | Low | High | Low | High |


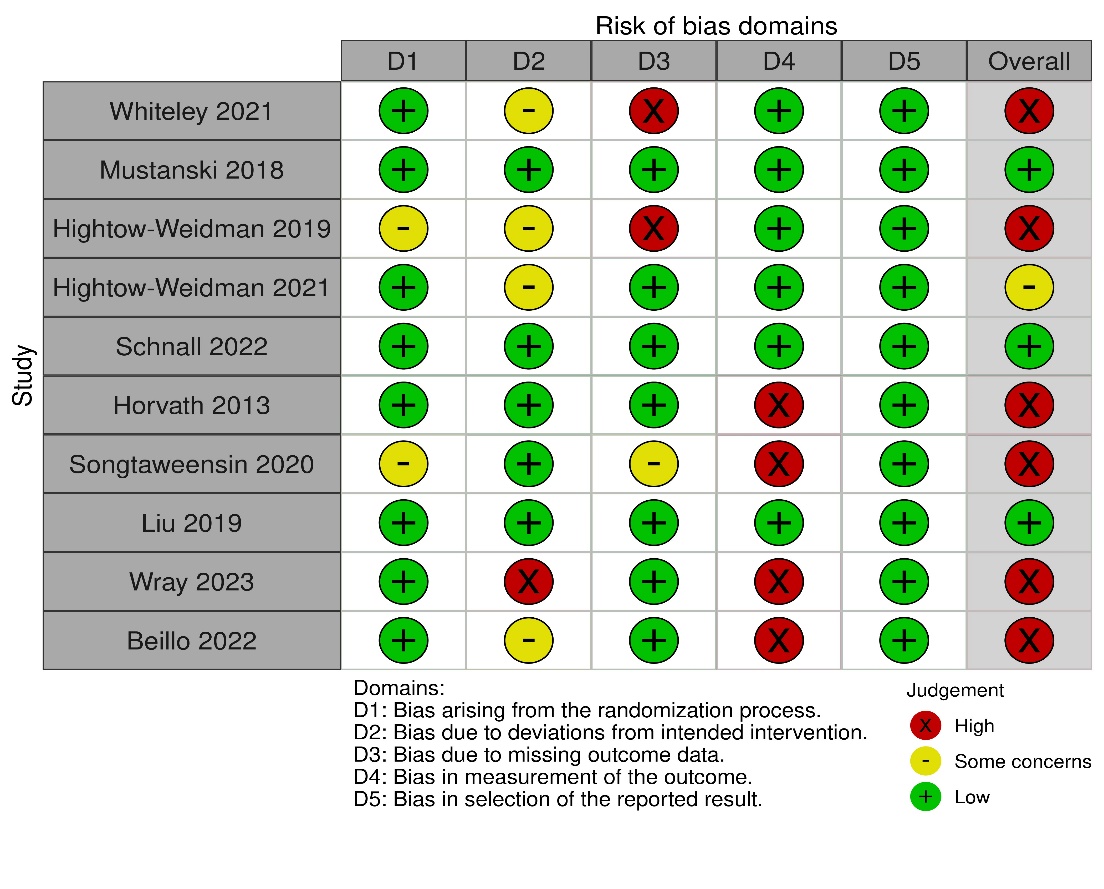


**Figure S4-1**. Risk of bias in RCTs. The risk of bias across RCTs included in the review was assessed using the revised Cochrane Collaboration’s Risk of Bias tool RoB2. RCTs received a low (green), high (red) or uncertain (yellow) risk of bias score for each of five domains.

**Table S4-2**. Risk of bias in included non-randomized studies by ROBINS-I tool

| Study | D1 | D2 | D3 | D4 | D5 | D6 | D7 | Overall |
| --- | --- | --- | --- | --- | --- | --- | --- | --- |
| Mitchell 2022 | Serious | Low | Low | Low | Low | Low | Low | Serious |
| Mitchell 2018 | Serious | Low | Low | Low | Low | Low | Low | Serious |
| McCoy 2018 | Critical | Serious | Low | Low | Serious | Moderate | Moderate | Critical |
| Garg 2020 | Critical | Low | Low | Low | Moderate | Critical | Low | Critical |
| Dworkin2019 | Critical | Low | Low | Low | Critical | Serious | Serious | Critical |
| Weitzman 2021 | Critical | Serious | Low | Low | Low | Serious | Moderate | Critical |


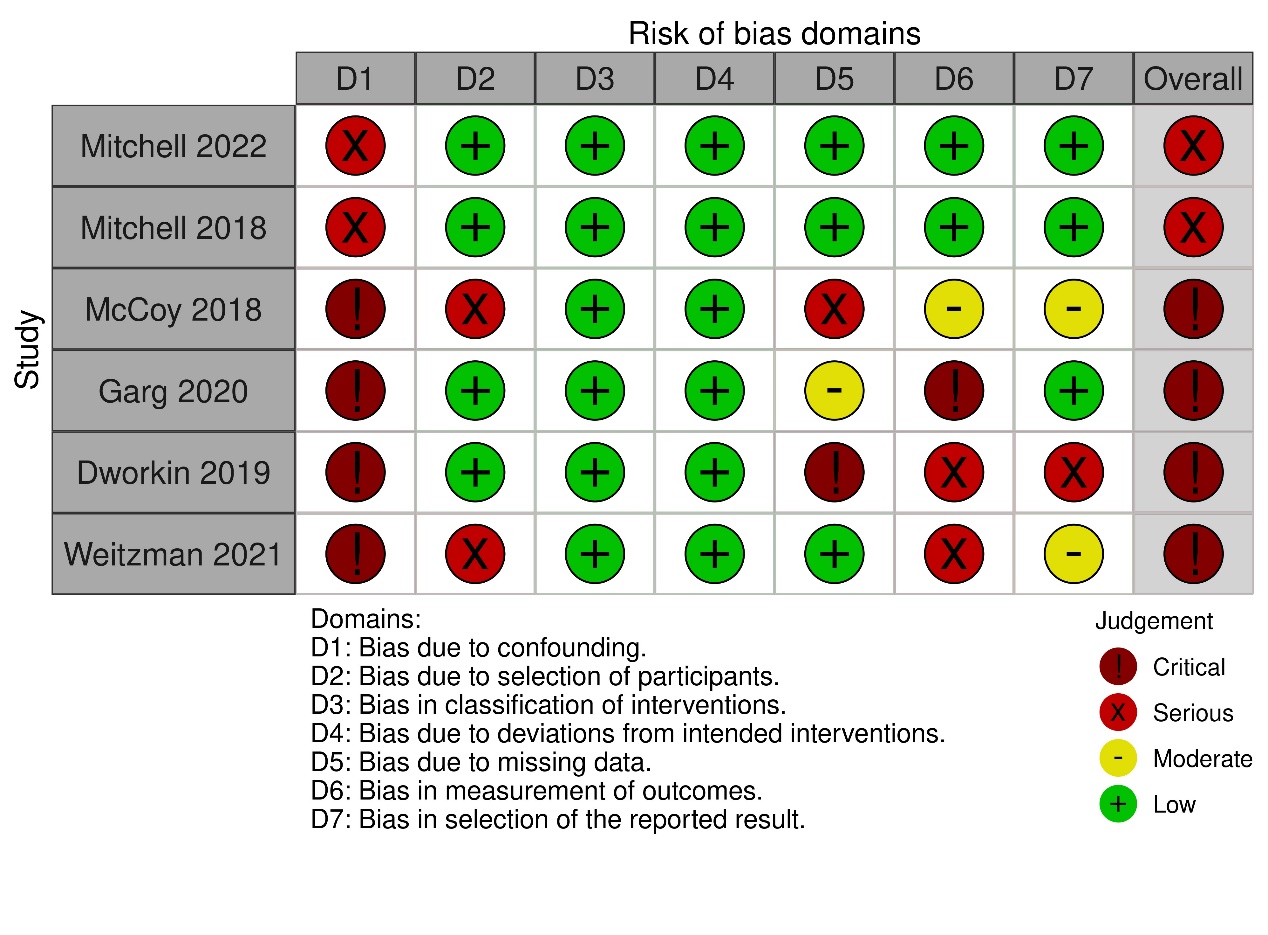


**Figure S4-2**. Risk of bias in non-randomized studies including cohort, case-control, controlled pre-post, and quasi-randomized studies. The risk of bias across each study included in the review was assessed using the Risk Of Bias In Non-randomized Studies-of Interventions ROBINS-I. Each study was judged as low (green), moderate (yellow), serious (bright red) or critical (dark red) risk of bias score for each of seven domains.
