# Supplementary material for: mHealth-Based Gamification Interventions Among Men Who Have Sex With Men in the HIV Prevention and Care Continuum: Systematic Review and Meta-Analysis
Source: JMIR Mhealth Uhealth. 2024 Apr 15;12:e49509. doi: 10.2196/49509 (PMC11034423; doi:10.2196/49509)
Supplement: Multimedia Appendix 4 [file mhealth-v12-e49509-s004.docx]

# Appendix 5. Study effects

**Table S5-1. List of studies in each outcome of interest**

| Outcome of interest | Non-RCTs | RCTs |
| --- | --- | --- |
| Risky sexual behaviors | Garg 2020; Besoain 2020; | Mustanski 2018*; Hightow-Weidman 2019; Schnall 2022*. |
| HIV testing | Shrestha 2023; McCoy 2018; Liu 2019; Andrade-Romo 2020; Bauermeister 2018. | Biello 2022. |
| PrEP uptake | Shrestha 2023; Liu 2019. | Biello 2022. |
| PrEP adherence | Mitchell 2022; Mitchell 2018; Mauka 2021; LeGrand 2018; Weitzman 2021. | Whiteley 2021*; Songtaweesin 2020*; Liu 2019*; Wray 2023*. |
| nPEP uptake | Luo 2022 | No study |
| nPEP adherence | No study | No study |
| ART initiation | No study | No study |
| ART adherence | Hightow-Weidman 2018; Dworkin 2019; Kim 2021. | Hightow-Weidman 2021; Hovath 2013. |

*: RCTs that were included in meta-analysis.

**Table S5-2**. Effects of gamification prevention on PrEP adherence (3-month)

| Study | RR | 95%CI | Weight (%) |
| --- | --- | --- | --- |
| Whiteley 2021 | 1.32719 | 0.809985, 2.17464 | 12.38 |
| Songtaweensin 2020 | 1.07284 | 0.798403, 1.44161 | 37.37 |
| Liu 2019 | 1.17759 | 0.910361, 1.52326 | 49.25 |
| D+L pooled RR | 1.15564 | 0.96467, 1.38441 |  |

Heterogeneity chi-squared = 0.57 (d.f.=2) p = 0.753

Estimate of between-study variance Tau-squared = 0.0000

Test of RR=1: z= 1.57 p = 0.116

**Table S5-3**. Effects of gamification prevention on PrEP adherence (6-month)

| Study | RR | 95%CI | Weight (%) |
| --- | --- | --- | --- |
| Whiteley 2021 | 2.22222 | 1.07644, 4.5876 | 16.24 |
| Songtaweensin 2020 | 1.11781 | 0.78420, 1.5933 | 43.18 |
| Liu 2019 | 1.48148 | 1.01757, 2.15688 | 40.58 |
| Wray 2023 | 1.002 | 0.874, 1.150 | 33.55 |
| D+L pooled RR | 1.280 | 0.892, 1.838 | 100.00 |

Heterogeneity chi-squared = 13.42 (d.f. = 3) p = 0.004

Estimate of between-study variance Tau-squared = 0.0965

Test of RR=1: z= 1.34 p = 0.181

**Table S5-4.** Effects of gamification intervention on number of anal condomless sex (3-month)

| Study | ES | 95%CI | Weight (%) |
| --- | --- | --- | --- |
| Schnall 2022 | 0.560 | 0.320, 0.980 | 38.26 |
| Mustanski 2018 | 0.668 | 0.430, 1.038 | 61.74 |
| D+L pooled ES | 0.624 | 0.442, 0.883 |  |

Heterogeneity chi-squared = 0.24 (d.f. = 1) p = 0.627

I-squared (variation in ES attributable to heterogeneity) = 0.0%

Estimate of between-study variance Tau-squared = 0.0000

Test of ES=1: z= 2.67 p = 0.008

**Table S5-5.** Effects of gamification intervention on number of anal condomless sex (6-month)

| Study | ES | 95%CI | Weight (%) |
| --- | --- | --- | --- |
| Schnall 2022 | 0.610 | 0.340, 1.094 | 81.17 |
| Mustanski 2018 | 1.410 | 0.340, 5.847 | 18.83 |
| D+L pooled ES | 0.714 | 0.376, 1.357 |  |

Heterogeneity chi-squared = 1.14 (d.f. = 1) p = 0.286

I-squared (variation in ES attributable to heterogeneity) = 12.3%

Estimate of between-study variance Tau-squared = 0.0432

Test of ES=1: z= 1.03 p = 0.304
