# Supplementary material for: mHealth-Based Gamification Interventions Among Men Who Have Sex With Men in the HIV Prevention and Care Continuum: Systematic Review and Meta-Analysis
Source: JMIR Mhealth Uhealth. 2024 Apr 15;12:e49509. doi: 10.2196/49509 (PMC11034423; doi:10.2196/49509)
Supplement: Multimedia Appendix 5 [file mhealth-v12-e49509-s005.docx]

# Appendix 6. Publication bias


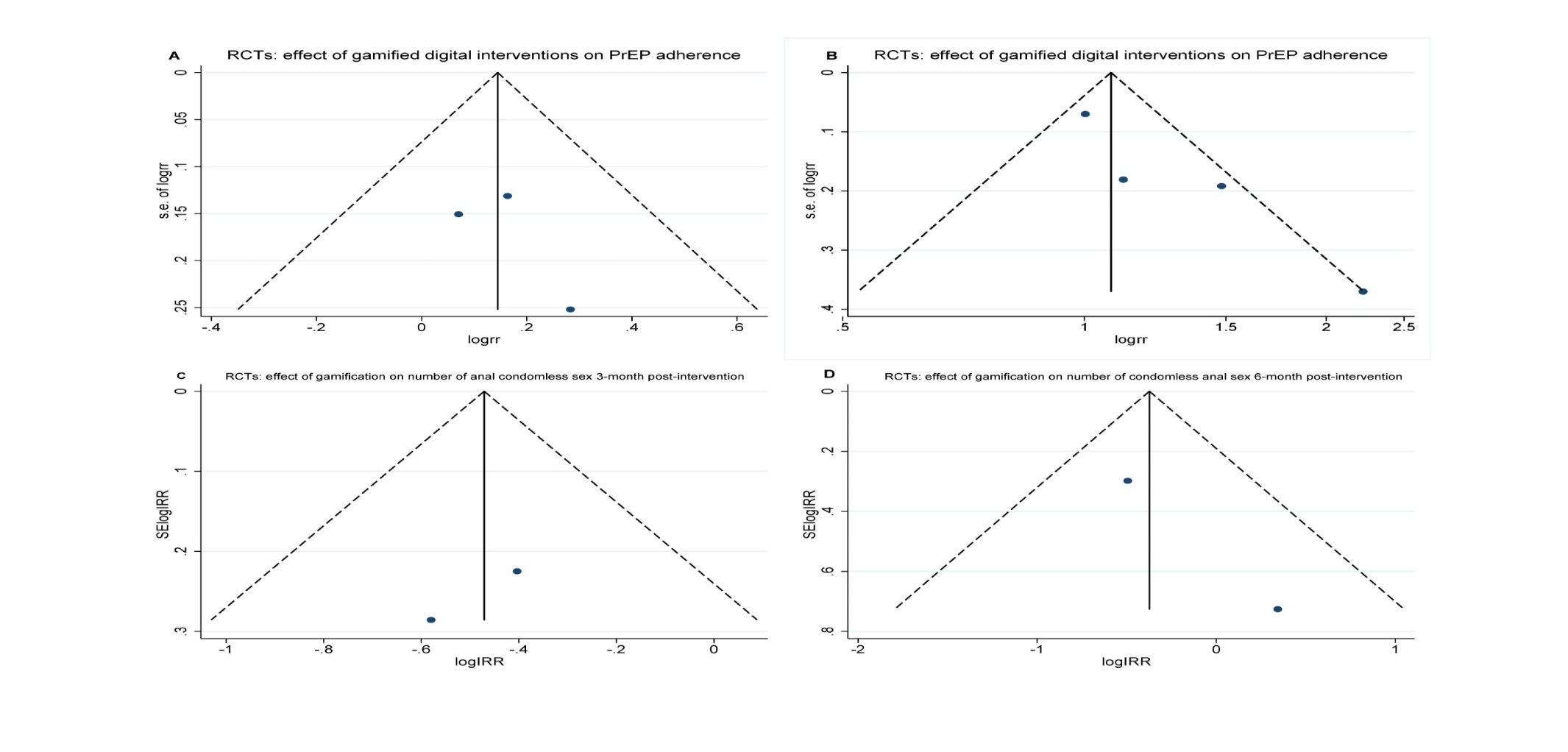
**Figure S6-1.** Potential publication bias was assessed in RCTs using funnel plots. Unadjusted effect estimates in each individual studies were plotted against their standard error. To identify potential publication bias, the egger test was used to assess funnel plot asymmetry.

(A) Funnel plot assessing publication bias in RCTs evaluating the effect of gamification on PrEP adherence 3-month post-intervention among MSM. Egger’s estimated bias coefficient: 1.099, p=0.572.

(B) Funnel plot assessing publication bias in RCTs evaluating the effect of gamification on PrEP adherence 6-month post-intervention among MSM. Egger’s estimated bias coefficient: 0.614 p=0.804.

(C) Funnel plot assessing publication bias in RCTs evaluating the effect of gamification on number of condomless anal sex at 3-month post-intervention among MSM. Egger’s estimated bias coefficient: -2.902, p=0.919.

(C) Funnel plot assessing publication bias in RCTs evaluating the effect of gamification on number of condomless anal sex at 6-month post-intervention among MSM. Egger’s estimated bias coefficient: 1.960, p=0.899.
